# APL‐like subset within *NPM1*‐mutated AML: A distinct immunophenotype correlating with early vascular complications

**DOI:** 10.1002/hem3.70307

**Published:** 2026-04-16

**Authors:** Francesco Mannelli, Francesca Crupi, Sara Bencini, Michaela Feuring, Gaia Ciolli, Matteo Piccini, Marco Frigeni, Raffaele Palmieri, Chiara Sartor, Barbara Scappini, Giacomo Gianfaldoni, Benedetta Peruzzi, Roberto Caporale, Antonio Scannella, Laura Fasano, Elisa Quinti, Andrea Pasquini, Jessica Caroprese, Leonardo Signori, Fabiana Pancani, Chiara Maccari, Tiziana Ottone, Daniela Spaeth, Antonio Curti, Maria Teresa Voso, Francesco Annunziato, Konstanze Döhner, Adriano Venditti, Francesco Buccisano, Alessandro Rambaldi, Paola Guglielmelli, Hartmut Döhner, Alessandro M. Vannucchi

**Affiliations:** ^1^ Dipartimento di Medicina Sperimentale e Clinica Università di Firenze Firenze Italy; ^2^ SOD Ematologia Università di Firenze, AOU Careggi Firenze Italy; ^3^ Centro Ricerca e Innovazione Malattie Mieloproliferative (CRIMM) AOU Careggi Firenze Italy; ^4^ Centro Diagnostico di Citofluorimetria e Immunoterapia AOU Careggi Firenze Italy; ^5^ Department of Internal Medicine III Ulm University Hospital Ulm Germany; ^6^ Azienda Socio‐Sanitaria Territoriale (ASST) Ospedale Papa Giovanni XXIII Bergamo Italy; ^7^ Dipartimento di Biomedicina e Prevenzione Università di Tor Vergata Roma Italy; ^8^ Fondazione Policlinico Tor Vergata Roma Italy; ^9^ Istituto di Ematologia “Seràgnoli” IRCCS Azienda Ospedaliero‐Universitaria di Bologna Bologna Italy; ^10^ Dipartimento di Scienze Mediche e Chirurgiche Università di Bologna Bologna Italy; ^11^ Department of Oncology and Hematology Università degli Studi di Milano Milano Italy

## Abstract

Among *NPM1*‐mutated acute myeloid leukemia (AML) (*NPM1* ^mut^), a distinct subtype has been described with an immunophenotypic profile resembling acute promyelocytic leukemia (APL‐like). In this retrospective multicenter study including 384 *NPM1* ^mut^ AML patients, we identified 95 (24.7%) cases exhibiting an APL‐like immunophenotype. This subset was characterized by significant abnormalities in coagulopathy markers (D‐dimer, D‐dimer/fibrinogen ratio, and disseminated intravascular coagulation [DIC] score). The cumulative incidence of vascular events at 30 days was significantly higher in the APL‐like group compared to the non‐APL‐like group (30.5% vs. 10.1%, P < 0.001). Notably, a higher cumulative incidence of early death due to vascular complications (within 30 days) was observed in the APL‐like group (6.3% vs. 0.35% in controls; P = 0.00015). In multivariate analysis, the APL‐like immunophenotype was the only significant factor associated with vascular‐related early death (hazard ratio [HR] = 19, P = 0.0063). There was a significantly higher rate of *IDH1/2* mutations in APL‐like (68.3%) compared to non‐APL‐like (18.3%, P < 0.001) cases. We validated these clinical and molecular findings in an independent validation cohort of 302 *NPM1* ^mut^ patients enrolled in the acute myeloid leukemia study group (AMLSG) 09‐09 clinical trial, which included the administration of all‐trans retinoic acid (ATRA) to all patients and a randomization for gemtuzumab ozogamicin. In this cohort, the APL‐like immunophenotype was associated with events occurring within the first 15 days but did not influence mortality, likely due to protocol‐driven patient selection. Our findings have important clinical implications that warrant the development of studies exploring disease‐tailored clinical measures to mitigate the risk of early vascular events, as in current APL management.

## INTRODUCTION

The therapeutic approach to patients with acute myeloid leukemia (AML), whether treated with intensive or low‐intensity regimens, relies on integrating multiple clinical and biological parameters, aiming to select the most appropriate strategy for achieving disease eradication or long‐term disease control.^1^

From a clinical standpoint, the initial phase can be particularly critical due to the potential for immediate, disease‐related complications, among which the most frequent are hyperleukocytosis, severe infections, coagulopathy, and extramedullary involvement. These manifestations may be life‐threatening and can significantly hinder the timely delivery of appropriate anti‐leukemic treatment. Therefore, the clinical stabilization of patients at AML onset is a crucial phase, serving as a prerequisite for initiating adequate disease‐directed therapeutic path.

Among clinical manifestations, coagulation disorders are frequent and potentially severe, and require prompt and appropriate management.^2^, ^3^ Acute promyelocytic leukemia (APL) is a well‐known AML subset typically associated with a complex coagulopathy, resulting in a relevant incidence of bleeding manifestations (12%–30%)^4^ and/or thrombosis (20%),^5^ with early mortality rates reaching up to 26% within the first 30 days from diagnosis.^6^ The well‐documented link between APL and coagulopathy has led to the development of specific measures aimed at mitigating its severity and the associated mortality, the first and most important of which is the immediate initiation of all‐trans retinoic acid (ATRA) upon clinical and/or morphological suspicion of APL.^7^

*NPM1*‐mutated (*NPM1* ^mut^) AML represents a distinct disease category in the current classifications,^8^, ^9^ although co‐mutation patterns can significantly influence clinical presentation, disease biology, and outcomes.^10^, ^11^ A distinct subtype of *NPM1* ^mut^ AML has recently been described as characterized by an immunophenotypic profile resembling that of APL and has therefore been termed APL‐like.^12^ This immunophenotype is specifically characterized by negativity for CD34 and HLA‐DR, and heterogeneous CD117 expression. Genetically, the APL‐like subtype is enriched in mutations in *IDH1/2* and *TET2*.^11^, ^12^

In the current study, we aimed to investigate the APL‐like subset within *NPM1* ^mut^ AML with special focus on early vascular events and markers of coagulopathy, taking advantage of a large, retrospective multicenter patient cohort. We also validated findings through a post hoc analysis of the prospective acute myeloid leukemia study group (AMLSG) 09‐09 clinical trial.^13^, ^14^

## METHODS

### Training cohort

In the training cohort, patients entering the study had a diagnosis of *NPM1* ^mut^ AML at study sites (Firenze, Bergamo, Bologna, and Roma Tor Vergata) according to morphological, immunophenotypic, and molecular criteria. Patients eligible for intensive chemotherapy were consecutively allocated to treatment based on the availability of a clinical trial and/or institutional treatment policies, as detailed in the Supplemental Data. The study was approved by the local institutional review board (IRB: project MYNERVA, approval 14560). Written informed consent was obtained from study patients in accordance with the Declaration of Helsinki.

### Laboratory parameters

Complete blood count, lactate dehydrogenase (LDH), and coagulation parameters, including prothrombin time (PT), international normalized ratio (INR), fibrinogen (FBG; Clauss method), and D‐dimer (DD; immunoturbidimetric method), were evaluated. Disseminated intravascular coagulation (DIC) score was assessed according to the 2018 revision of the ISTH DIC‐score.^15^ An overt DIC was defined by a DIC score ≥ 4.

### Flow cytometry

Technical details regarding sample handling, reagents, acquisition, and analysis are provided in the Supplemental File. For data analysis in the training cohort, Infinicyt (Cytognos SL, Salamanca, Spain) software was used. For analysis in the validation cohort, flow cytometry data were extracted from the database of the clinical trial. The analytical strategy was performed according to shared operative procedures. Specifically, the blast cell compartment was identified at diagnosis according to the expression of CD45 and side scatter (SCC) signal. A number of phenotypic parameters were expressed as a percentage of positive cells within the blast cell population. Antigen expression was determined using the autofluorescence as a reference control. APL‐like definition relied on negativity for CD34 and HLA‐DR according to conventional thresholds (i.e., less than 20% on blast cell population) (Figure S1).

### Molecular methods

*NPM1*, *FLT3*, and *CEBPA* mutations were analyzed according to previously described methods.^16^, ^17^ Further details are reported in Supplemental Data. A subgroup of patients was characterized by Next‐Generation deep amplicon sequencing using the Ion Torrent platform (ThermoFisher Scientific, Waltham, Massachusetts, USA) to investigate a custom panel of a series of 40 genes. Based on targeted PCR and NGS data, the relative gene status was available for *IDH1* (*n* = 229), *IDH2* (*n* = 229), *DNMT3A* (*n* = 201), *TET2* (*n* = 177), and *FLT3* (*n* = 379); *CEBPA*, *PTPN11*, *STAG2*, *WT1*, *NRAS*, *KRAS*, *HRAS*, *PRPF8*, *BCOR*, *JAK2*, and *SRSF2* (*n* = 122); *TP53*, *SH2B3*, *NF1*, *U2AF1*, *CSF3R*, *RUNX1*, *ASXL1*, *KIT*, *MPL*, *SF3B1*, *MYD88*, *GATA2*, *IKZF1*, *BRAF*, *EZH2*, *ABL1*, *CBL*, *ETV6*, *RB1*, *SETBP1*, *CALR*, *ZRSR2*, *BCOR*, and *PHF6* (*n* = 95). For genes with known mutational hotspots, only those regions were amplified, otherwise all coding exons were sequenced. Sequence alignment and filtering were performed using NextGENe version 2.4.2.1 (SoftGenetics, Pennsylvania, USA).

### Validation cohort

The prospective, multicenter randomized AMLSG 09‐09 clinical trial^13^, ^14^, ^18^ served as an external validation dataset to confirm findings on the incidence of vascular events, correlation with molecular data, and patient outcomes. AMLSG 09‐09 was an open‐label, Phase 3 trial that randomly assigned *NPM1* ^mut^ AML patients in a 1:1 ratio to receive intensive chemotherapy and ATRA, with or without gemtuzumab ozogamicin (GO) (Clinicaltrial.gov NCT00893399).

### Definitions

Complete remission (CR), disease‐free survival (DFS), event‐free survival (EFS), and overall survival (OS) were defined according to standard criteria.^1^ Vascular events were reported according to the WHO bleeding scale^19^, ^20^, ^21^ for hemorrhagic events and Common Terminology Criteria for Adverse Events (CTCAE) version 5.0 (Bethesda, MD: National Institutes of Health, U.S. Department of Health and Human Services; 2017. Available from: https://ctep.cancer.gov/protocolDevelopment/electronic_applications/ctc.htm) for thromboembolic events. Clinical criteria for the definition of thromboembolic events were represented by deep vein thrombosis or arterial thrombosis at any site, diagnosed by Doppler ultrasonography, computed tomography, or magnetic resonance imaging. For hemorrhagic events, clinically irrelevant manifestations (i.e., Grade 1 skin manifestations) were excluded from the analysis. In case of multiple events in a single patient, the first event occurring within 30 days and having the most clinically relevant impact was considered. Early mortality rate was defined as death for any cause within 30 days from diagnosis and further detailed for cause as attributed to one among pre‐specified categories (disease progression, vascular complications, infectious complications, and others). As regards prognostic stratification in the training dataset, the criteria used for the therapeutic decision‐making across different time periods, and particularly allocation to hematopoietic stem cell transplantation (HSCT), are specified in the Supplemental File.

### Statistical methods

Pairwise comparisons of patient characteristics between groups, as defined by the APL‐like immunophenotype, were performed using the Mann–Whitney test or the Kruskal–Wallis test for continuous variables and the Pearson's chi‐squared test or Fisher's exact test for categorical variables. Survival was estimated using the Kaplan–Meier method, and long‐term outcomes were compared using the log‐rank test. The Cox proportional‐hazards model was applied to estimate hazard ratios (HRs) with 95% confidence intervals (CIs) for DFS, EFS, and OS in both univariate and multivariate contexts. The Gray's test was applied to assess differences in the cumulative incidence of mortality due to vascular versus other causes between groups. Median follow‐up time was estimated by reversing the codes for the censoring indicator in a Kaplan–Meier analysis. To rule out the impact of HSCT, we censored patients who received HSCT at the date of transplant in a further analysis. A propensity score‐matched analysis was carried out selecting an *NPM1* wild‐type (wt) control group. Propensity scores were matched in a 1:1 ratio using the nearest‐neighbor algorithm. All P values were two‐sided, and a 5% significance level was set. All statistical analyses were performed using R version 4.4.2.

## RESULTS

### Analysis of baseline clinical and laboratory characteristics

From April 2007 to October 2024, 384 patients with *NPM1* ^mut^ AML met the inclusion criteria at project study sites, of whom 95 (24.7%) were classified as APL‐like. Regarding baseline characteristics, APL‐like patients were older (66.4 vs. 58.6 years, P < 0.001) and showed lower platelet count (40 vs. 57 × 10^9^/L, P = 0.006) than non‐APL‐like patients (Table 1). APL‐like patients showed marked alterations in coagulation parameters compared to non‐APL‐like patients. Specifically, both DD (5998 ng/mL) and DD/FBG ratio (16.8) were significantly higher than in non‐APL‐like (2219 ng/mL, P = 0.001, and 5.1, P < 0.001, respectively). Furthermore, the median DIC score was significantly higher in the APL‐like group than in the non‐APL‐like counterpart (4 vs. 3, P = 0.004), and a larger proportion of patients had an overt (≥4) DIC score (57.9% vs. 35.9%, P = 0.005). As regards treatment in the overall cohort, 304 (79.2%) patients received intensive chemotherapy, 46 (12.0%) received low‐intensity treatments, and 34 (8.8%) received supportive care only. A significant difference in delivery of induction treatment was observed between APL‐like and non‐APL‐like groups, either considering any induction (80.0% vs. 94.4%, respectively; P < 0.001) or intensive treatment only (65.3% vs. 83.7%, respectively; P < 0.001), possibly due to significantly older age for the former group (Table 1).

**Table 1:** Clinical, molecular, and treatment characteristics of the learning cohort.

	Overall	Non‐APL‐Like	APL‐Like	P
Total, *n* (%)	384	289 (75.3%)	95 (24.7%)	
Age (years), median (range)	60.5 (51–68)	58.6 (48–66.1)	66.4 (58.4–73.1)	**<0.001**
Male, *n* (%)	175 (45.6%)	124 (42.9%)	51 (53.7%)	0.075
Female, *n* (%)	209 (54.4%)	165 (57.1%)	44 (46.3%)	
WBC count (×10^9^/L), median (range)	35.1 (10.5–87.95)	35.0 (10.5–85.7)	42.0 (11.3–115.4)	0.307
Hb (g/dL), median (range)	9.0 (7.7–10.2)	8.9 (7.7–10.3)	8.8 (7.5–9.9)	0.409
Plt (×10^9^/L), median (range)	52 (31–90)	57 (34–90)	40 (24–81)	**0.006**
LDH (U/L), median (range)	616 (385–1021)	691 (428–1171)	463 (341–648)	**<0.001**
INR ≥ 1.5, *n* (%)	51 (14.6%)	31 (11.9%)	20 (22.7%)	**0.022**
FBG ≤ 150 (mg/dL), *n* (%)	35 (10.4%)	22 (8.7%)	13 (15.5%)	0.097
DD (ng/mL), median (range)	2938 (1023–11,132)	2219 (921–6731)	5998 (2339–32,238)	**0.001**
DD/FBG, *n* (range)	6.9 (2.1–35.1)	5.1 (1.8–19.0)	16.8 (3.4–191.7)	**<0.001**
Vascular events, *n* (%)	59 (15.4%)	30 (10.5%)	29 (30.5%)	**<0.001**
Bleeding (BC), *n* (%)	45 (11.7%)	26 (9.0%)	19 (20.0%)	**<0.001**
Thrombotic (TC), *n* (%)	14 (3.7%)	4 (1.4%)	10 (10.5%)	**<0.001**
Grading WHO BC, *n* (%)				
G1–G2	31 (8.1%)	20 (6.9%)	11 (11.6%)	0.19
G3–G5	14 (3.6%)	6 (2.1%)	8 (8.4%)	**0.0085**
CTCAE TC, *n* (%)				
G1–G2	5 (1.3%)	2 (0.7%)	3 (3.2%)	0.099
G3–G5	9 (2.3%)	2 (0.7%)	7 (7.4%)	**0.0011**
DIC score, median (range)	3 (2–5)	3 (2–5)	4 (2–6)	**0.004**
DIC score ≥ 4, *n* (%)	93 (41.5%)	60 (35.9%)	33 (57.9%)	**0.005**
*NPM1* mutation type, available, *n* (%)	335	254	81	
A	279 (83.3%)	210 (82.7%)	69 (85.2%)	0.73
B	20 (6.0%)	19 (7.5%)	1 (1.2%)	0.22
D	11 (3.3%)	6 (2.4%)	5 (6.2%)	0.14
Other	25 (7.4%)	18 (7.0%)	7 (8.6%)	0.66
*NPM1* mutation type, not available, *n* (%)	49	35	14	
*FLT3* status, available, *n* (%)	379	285	94	
Any	195 (51.5%)	146 (51.2%)	49 (52.1%)	0.906
TKD	40 (10.6%)	31 (10.9%)	9 (9.6%)	0.847
ITD	169 (44.6%)	127 (43.9%)	44 (46.8%)	0.634
*IDH* status, available, *n* (%)	229	169	60	
Any	72 (31.4%)	31 (18.3%)	41 (68.3%)	**<0.001**
*IDH1*	32 (14.0%)	17 (10.0%)	15 (25.0%)	**0.008**
*IDH2*	40 (17.5%)	14 (8.3%)	26 (43.3%)	**<0.001**
*TET2* status, available, *n* (%)	177	129	48	
Mutated	39 (22.0%)	28 (21.7%)	11 (22.9%)	0.841
*DNMT3A* status, available, *n* (%)	201	152	49	**0.032**
Mutated	98 (48.8%)	81 (53.3%)	17 (34.7%)	
Induction treatment, *n* (%)	349 (90.9%)	273 (94.4%)	76 (80.0%)	**<0.001**
Intensive	304 (79.2%)	242 (83.7%)	62 (65.3%)	**<0.001**
Non‐intensive	45 (11.7%)	31 (10.7%)	14 (14.7%)	
BSC, *n* (%)	35 (9.1%)	16 (5.5%)	19 (20.0%)	
CR rate in intensively treated, *n* (%)				
CR after first cycle	245 (80.6%)	194 (80.2%)	51 (82.3%)	0.857
CR any time	267 (87.8%)	209 (86.4%)	58 (93.5%)	0.189
HSCT, *n* (%)	139 (36.2%)	116 (40.1%)	23 (24.2%)	**0.005**

*Note*: Differences between treatment groups were evaluated using the Mann–Whitney test for continuous variables and Fisher's exact tests or *χ* ^2^ for categorical variables. Values in bold are statistically significant (P < 0.05).

Abbreviations: APL, acute promyelocytic leukemia; BSC, best supportive care; CR, complete remission; CTCAE, Common Terminology Criteria for Adverse Events; DD, D‐dimer; DIC, disseminated intravascular coagulation; FBG, fibrinogen; Hb, hemoglobin; HSCT, hematopoietic stem cell transplantation; INR, international normalized ratio; ITD, internal tandem duplication; LDH, lactate dehydrogenase; Plt, platelet count; TKD, tyrosine kinase domain; WBC, white blood cells; WHO, World Health Organization.

### Molecular genetics

*NPM1* mutation type was available for 335 (87.2%) of 384 patients. In the remaining 49 (12.8%) patients, the definition of *NPM1*‐mutated AML relied on the demonstration of the cytoplasmatic localization of the protein by immunohistochemistry on bone marrow (BM) biopsy.^22^ No significant difference was observed in the distribution of *NPM1* mutation type according to the APL‐like immunophenotype (Table 1).

In the overall series, the most commonly co‐mutated genes were *FLT3* (internal tandem duplication [ITD] 44.6%; tyrosine kinase domain [TKD] 10.6%), *DNMT3A* (48.8%), *IDH1* (14.0%), *IDH2* (17.5%), and *TET2* (22.0%). No difference in the frequency of *FLT3* mutations was observed between APL‐like (51.2%) and non‐APL‐like (52.1%, P = 0.906). A significantly higher rate of mutations in *IDH1* (25.0%) and *IDH2* (43.3%) genes occurred in APL‐like as compared to non‐APL‐like (10.0%, P = 0.008; 8.3%, P < 0.001, respectively) group (Table 1). Conversely, *DNMT3A* mutations were significantly enriched among non‐APL‐like patients (53.3% vs. 34.7%, P = 0.032). No difference in the incidence of *TET2* mutations was observed (21.7% vs. 22.9%, P = 0.841).

### Incidence of vascular events, early mortality, and long‐term outcome

**Figure 1:**
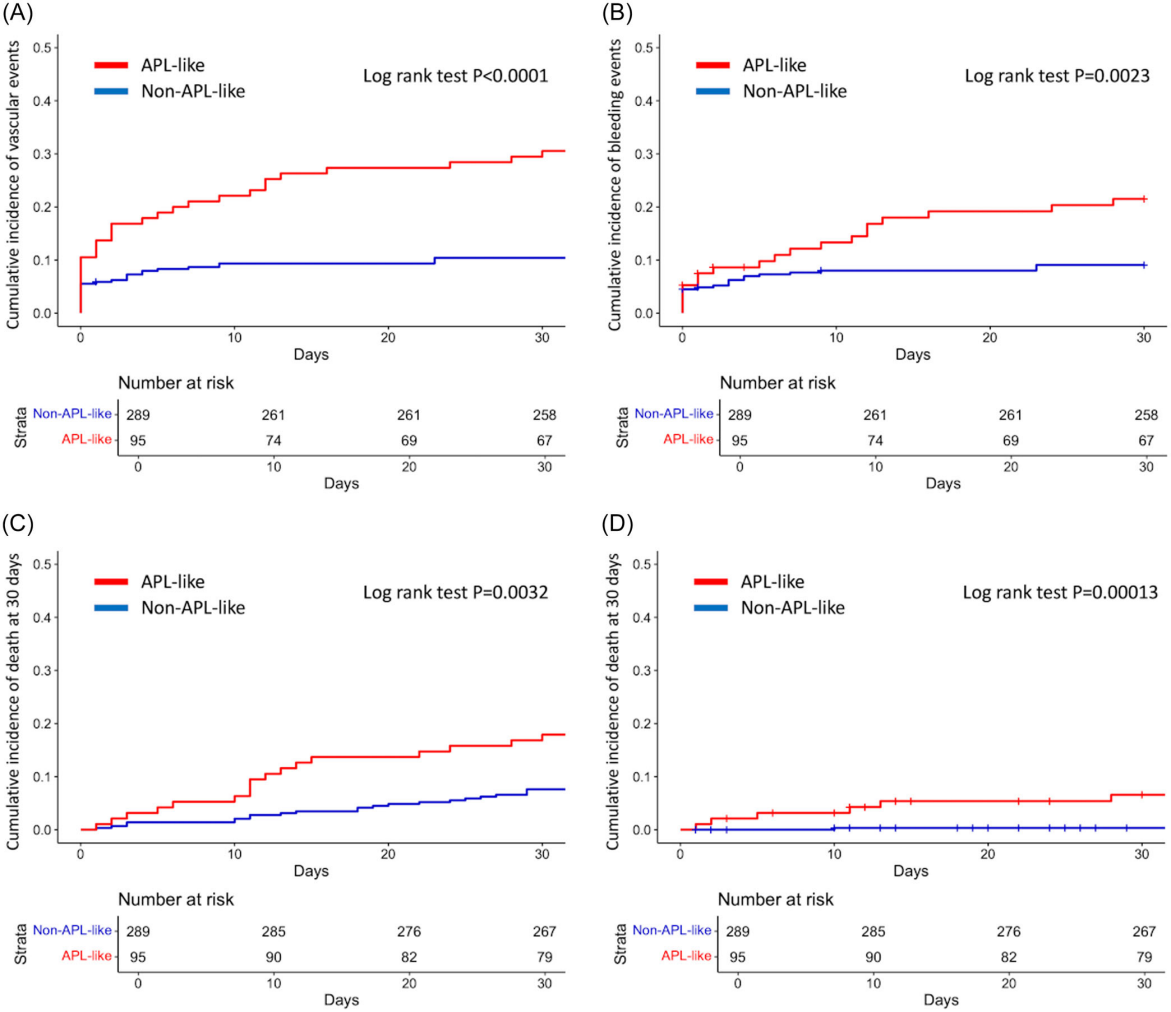
**Cumulative incidence of events according to acute promyelocytic leukemia (APL)‐like signature in the training cohort.** Plots of cumulative incidence of events at 30 days: **(A)** vascular events (gathering bleeding and thrombotic); **(B)** bleeding events. Plots of cumulative incidence of death at 30 days: **(C)** due to any cause; **(D)** due to vascular events. The curves of patients with APL‐like signature are depicted in red; the curves of non‐APL‐like patients are depicted in blue. The curves were created using R software.

Regarding clinically relevant vascular complications, 59 events occurred in the entire cohort, of which 45 (76.3%) were hemorrhagic and 14 (23.7%) thrombotic. The cumulative incidence of vascular events at 30 days was significantly higher in the APL‐like group (30.5%) compared to non‐APL‐like group (10.1%, P < 0.001) (Figure 1A), and encompassing both bleeding (20.0% vs. 9.0%, P < 0.0001) and thrombotic (10.5% vs. 1.4%, P < 0.0001) events. Notably, we observed a significant enrichment of high‐grade (Grades 3–5) events, both bleeding (P = 0.0085) and thrombotic (P = 0.0011), in the APL‐like group (Table 1).

*NPM1* mutation type (A vs. non‐A) had no effect on vascular events (P = 0.67; Figure S2). In multivariate analysis, including age (</≥60 years), WBC count (</≥50 × 10^9^/L), and platelet count (</≥50 × 10^9^/L), the APL‐like immunophenotype remained an independent predictive factor of vascular complications (HR = 3.0; 95% CI, 1.8–4.9; P < 0.001) along with leukocytosis (P = 0.011) (Table S1). When only hemorrhagic events were considered, APL‐like immunophenotype confirmed its impact on the cumulative incidence (P = 0.0023; Figure 1B), and it was the only significant factor in multivariate analysis (P = 0.0061) (Table S1).

Notably, in a comprehensive model including coagulation parameters (INR </≥ 1.5, FBG </≥ 150 mg/dL, and DIC score </≥ 4) and platelet count (</≥50 × 10^9^/L), the APL‐like immunophenotype retained its independent prognostic value (HR = 2.2; 95% CI, 1.1–4.4; P = 0.017), together with DIC score (HR = 2.7; 95% CI, 1.1–6.3; P = 0.024) while other parameters lost statistical significance Table S2.

Of note, a significantly higher cumulative incidence of early death at 30 days was observed in APL‐like (17.9%) compared to non‐APL‐like patients (7.6%, P = 0.0032) (Figure 1C). In multivariate analysis, age (P = 0.0001), WBC (P = 0.03), and platelet count (P = 0.002) were independent factors for early mortality, whereas APL‐like immunophenotype lost its significance (P = 0.26) (Table S3). However, when the analysis was restricted to early deaths due to vascular complications, APL‐like immunophenotype was significant in univariate analysis (Figure 1D; P = 0.00013) and remained the only significant factor (HR = 19; 95% CI, 2.3–158; P = 0.0063) in multivariate analysis (Table S3). To further confirm this correlation, we ran a competing event model and highlighted a significant difference in deaths attributed to vascular events between the APL‐like group (6.3%) and the non‐APL‐like group (0.35%, Gray test P = 0.00015), corresponding to a risk almost 20 times higher (Figure S3). No significant difference was found for death due to other causes (P = 0.18) (Figure S3). The majority (6 of 7) of fatal vascular events were intracranial bleedings, occurring predominantly in the APL‐like group (Table S4). Notably, these events occurred very early after diagnosis (i.e., within 15 days from referral) and represented the key reason for preventing the initiation of induction treatment in five out of the seven patients.

As APL‐like group was enriched in *IDH1* and *IDH2* mutations, we investigated the impact of the mutated status on vascular events. In univariate analysis, *IDH1* mutations (P = 0.11) did not show a significant influence, whereas *IDH2* (P = 0.024) and aggregate *IDH1/2* mutations correlated with a higher incidence of vascular events (Figure S4). In a multivariate model including both their status and the APL‐like immunophenotype, *IDH1/2* mutations lost statistical significance (HR = 1.7; P = 0.13), while the APL‐like subset retained its independent prognostic value (HR = 2.3; 95% CI, 1.1–4.5; P = 0.020).

**Figure 2:**
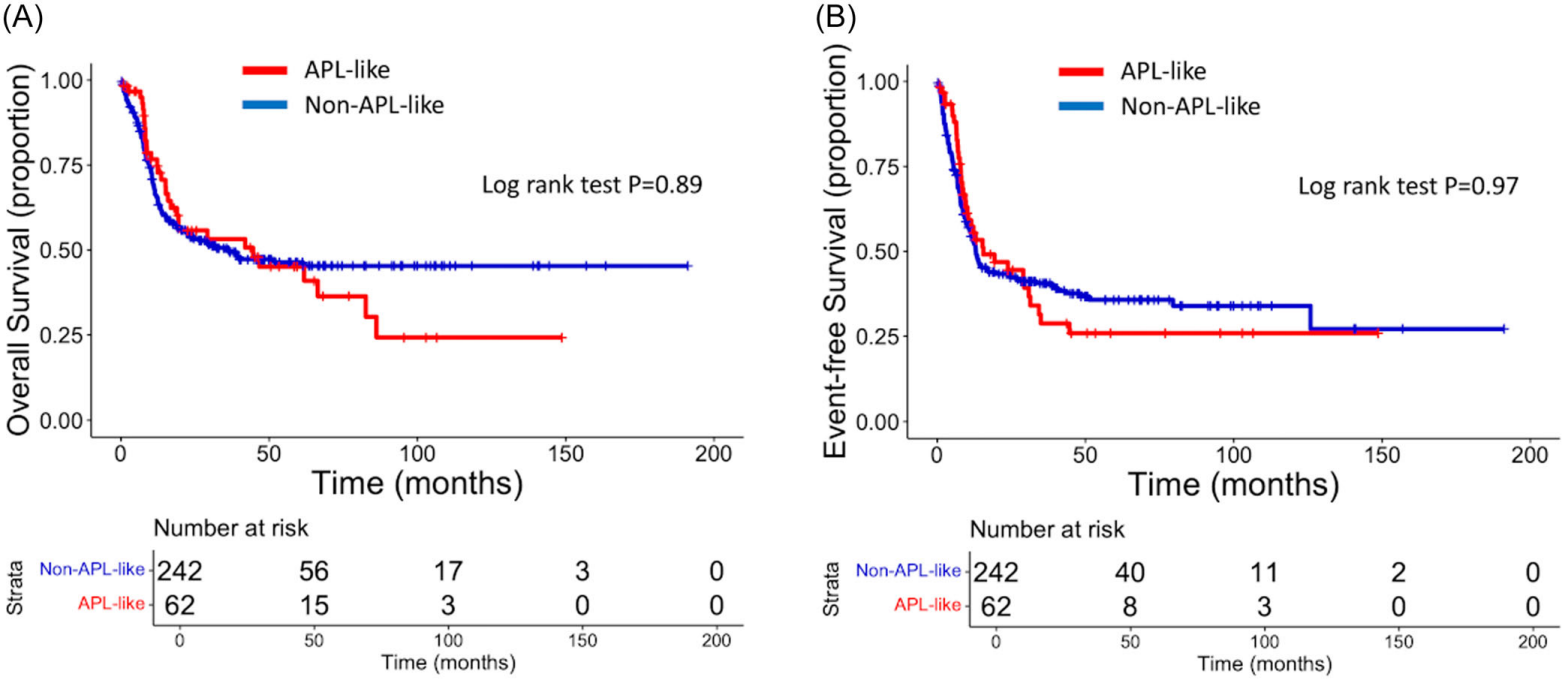
**Long‐term outcome according to acute promyelocytic leukemia (APL)‐like immunophenotype in the training cohort.** Kaplan–Meier curves of **(A)** overall and **(B)** event‐free survival show superimposable long‐term outcome. The curves of patients with APL‐like signature are depicted in red; the curves of non‐APL‐like patients are depicted in blue. The curves were created using R software.

Among patients receiving intensive chemotherapy, the incidence of vascular events was significantly higher in the APL‐like group compared with controls (P = 0.0067, Figure S5), while there was no difference in the cumulative incidence of early death due to vascular events (2.8% vs. 1.6%, respectively; P = 0.58). Also, there was no significant difference in CR rate (93.5% vs. 86.4%; P = 0.189), EFS (15.5 vs. 13.1 months; P = 0.97), and OS (44.5 vs. 36.1 months; P = 0.89) between APL‐like and non‐APL‐like patients, respectively (Figure 2).

### Propensity score matching

To confirm the impact of the APL‐like immunophenotype on coagulopathy and vascular complications also outside the setting of *NPM1* ^mut^ AML, we carried out a secondary analysis comparing the 95 APL‐like patients to a different control group, namely an unselected set of 100 patients with *NPM1* wt (*NPM1* ^wt^) AML consecutively observed at our Centre between 2016 and 2024. As regards baseline features, APL‐like patients were significantly older and exhibited higher WBC and lower platelet counts than the *NPM1* ^wt^ cohort (Table S5). Compared to the *NPM1* ^wt^ group, the APL‐like group showed significantly higher incidence of vascular complications (30.5% vs. 6.0%, P < 0.001), DD/FBG ratio (median 18.17 vs. 2.20, P < 0.001), and frequency of overt DIC scores (57.9% vs. 16.0%, P < 0.001).

To mitigate the influence of baseline characteristics on the coagulation abnormalities, we ran a propensity score matching to adjust the two cohorts for age, WBC, and platelet counts. After matching for age and platelets, the APL‐like group maintained significantly altered coagulation parameters (DD, DD/FBG, and DIC score) and a higher incidence of vascular events (Table S6).

Due to a marked pre‐matching unbalance in WBC between APL‐like and *NPM1* ^wt^ groups (42.0 vs. 4.8 × 10^9^/L, respectively), the matching method selected a limited number of patients for each group (16 vs. 18). We still noticed differences in vascular complications (25.0% vs. 5.6%; P = 0.164), DD/FBG ratio (6.1 vs. 1.55; P = 0.162), and overt DIC score (27.3 vs. 16.7; P = 0.646) not reaching statistical significance (Table S6).

### Results from validation dataset of AMLSG 09‐09 clinical trial

To validate findings from the training series, we interrogated the dataset of the AMLSG 09‐09 clinical trial, which enrolled 600 *NPM1* ^mut^ patients between May 2010 and September 2017. Clinical and laboratory data were extracted for 302 patients who had immunophenotypic characterization available. We compared training and validation cohorts for baseline clinical and laboratory characteristics (Table S7). Compared to the training cohort, patients in the validation cohort were younger (median age 53.6 vs. 60.5 years, P < 0.001), had lower WBC (21.8 vs. 35.1 × 10^9^/L, P < 0.001), and higher platelet (77 vs. 52 × 10^9^/L, P < 0.001) counts. The difference in age is consistent with patient selection for a clinical trial, which provided intensive chemotherapy for all patients. Moreover, *FLT3*‐ITD mutations were significantly less frequent in the validation cohort (18.2% vs. 44.6%, P < 0.001). This finding likely accounts for the difference in WBC and can be ascribed to the recruitment of patients exhibiting mutated *NPM1* in combination with *FLT3*‐ITD in the AMLSG 16‐10 study,^23^ open for accrual concomitantly to the AMLSG 09‐09 trial between June 2012 and May 2016.

Of the 302 patients available, 72 (23.9%) cases were classified as APL‐like. The proportion of APL‐like patients among *NPM1* ^mut^ was thus superimposable (24.7% and 23.9%) in the two cohorts. APL‐like patients were older (55.7 vs. 52.7 years, P = 0.030) and had lower platelet count (65 vs. 78 × 10^9^/L, P = 0.015) compared to non‐APL‐like patients, in analogy to what was observed in the training set. Baseline patient characteristics are detailed in Table 2.

**Table 2:** Clinical, molecular, and treatment characteristics in the acute myeloid leukemia study group (AMLSG) 09‐09 cohort of patients.

	**Overall**	**Non‐APL‐Like**	**APL‐Like**	**P**
Total, *n* (%)	302	230 (76.1%)	72 (23.8%)	
Age (years), median (range)	53.6 (45.7–58.7)	52.7 (45.6–58.1)	55.6 (49.9–60.5)	**0.030**
Male, *n* (%)	136 (45%)	101 (43.9%)	35 (48.6%)	0.5
Female, *n* (%)	166 (55%)	129 (56.1%)	37 (51.4%)	
WBC (×10^9^/L), median (range)	21.8 (6.12–55.4)	20.9 (7.25–54.25)	24.6 (4.2–62.9)	0.767
Hb (g/dL), median (range)	9.0 (7.9–10.4)	8.9 (7.9–10.1)	9.7 (8.1–11.3)	**0.009**
Plt (×10^9^/L), median (range)	77 (47–126)	78 (52–136)	65 (37–106)	**0.015**
Blasts (%), median (range)				
BM	71 (42–88)	64 (40–85)	80 (70–90)	<**0.001**
PB	20 (4–52)	14 (3–42)	58 (17–86)	<**0.001**
LDH (U/L), median (range)	444.5 (319–688.5)	464.5 (321.7–730.2)	398 (272–544.2)	**0.019**
Median time to vascular complications (days), median (range)	17 (11–20)	17.5 (13–21)	12 (7.5–18)	**0.020**
Bleeding, *n* (%)	52 (17.2%)	29 (12.6%)	23 (31.9%)	**0.0005**
Thrombotic, *n* (%)	6 (2.0%)	4 (1.7%)	2 (2.8%)	0.631
WHO/CTCAE grading, *n* (%)				
G1–G2	25 (8.3%)	19 (8.3%)	6 (8.3%)	1.0
G3–G5	56 (18.5%)	37 (16.1%)	19 (26.4%)	0.056
*NPM1* mutation type, available, *n* (%)	302	230	72	
A	216 (71.5%)	159 (69.1%)	57 (79.2%)	0.10
B	28 (9.3%)	23 (10.0%)	5 (6.9%)	0.63
D	23 (7.6%)	21 (9.1%)	2 (2.8%)	0.13
Other	35 (11.6%)	27 (11.8%)	8 (11.1%)	1.0
*FLT3* any, *n* (%)	91 (30.1%)	67 (29.1%)	24 (33.3%)	0.556
TKD	40 (14.1%)	33 (14.3%)	7 (9.7%)	0.425
ITD	55 (18.2%)	37 (16.1%)	18 (25.0%)	0.114
*IDH* any, *n* (%)	109 (36.6%)	68 (29.8%)	41 (58.6%)	**<0.001**
*IDH1*	56 (18.8%)	40 (17.5%)	16 (22.9%)	0.381
*IDH2*	59 (19.8%)	34 (14.9%)	25 (35.7%)	**0.0003**
*TET2*, *n* (%)	46 (15.4%)	29 (12.7%)	17 (24.3%)	**0.0237**
*DNMT3A*, *n* (%)	167 (55.3%)	151 (65.7%)	16 (22.2%)	**<0.001**
Treatment, *n* (%)				
CHT + ATRA	153 (51.0%)	115 (50.2%)	38 (53.5%)	0.684
CHT + ATRA + GO	147 (49.0%)	114 (49.2%)	33 (46.5%)	
CR1, *n* (%)	267 (92.7%)	204 (92.7%)	63 (92.6%)	1.0
HSCT, *n* (%)	26 (8.6%)	19 (8.3%)	7 (9.7%)	0.639

*Note*: Differences between treatment groups were evaluated using the Mann–Whitney test for continuous variables and Fisher's exact tests or *χ* ^2^ for categorical variables. Values in bold are statistically significant (P < 0.05).

Abbreviations: ATRA, all‐trans retinoic acid; BM, bone marrow; CHT, chemotherapy according to “3 + 7” course; CR, complete remission; CTCAE, Common Terminology Criteria for Adverse Events; GO, gemtuzumab ozogamicin; Hb, hemoglobin; HSCT, hematopoietic stem cell transplantation; ITD, internal tandem duplication; LDH, lactate dehydrogenase; PB, peripheral blood; Plt, platelet count; TKD, tyrosine kinase domain; WBC, white blood cells; WHO, World Health Organization.

**Figure 3:**
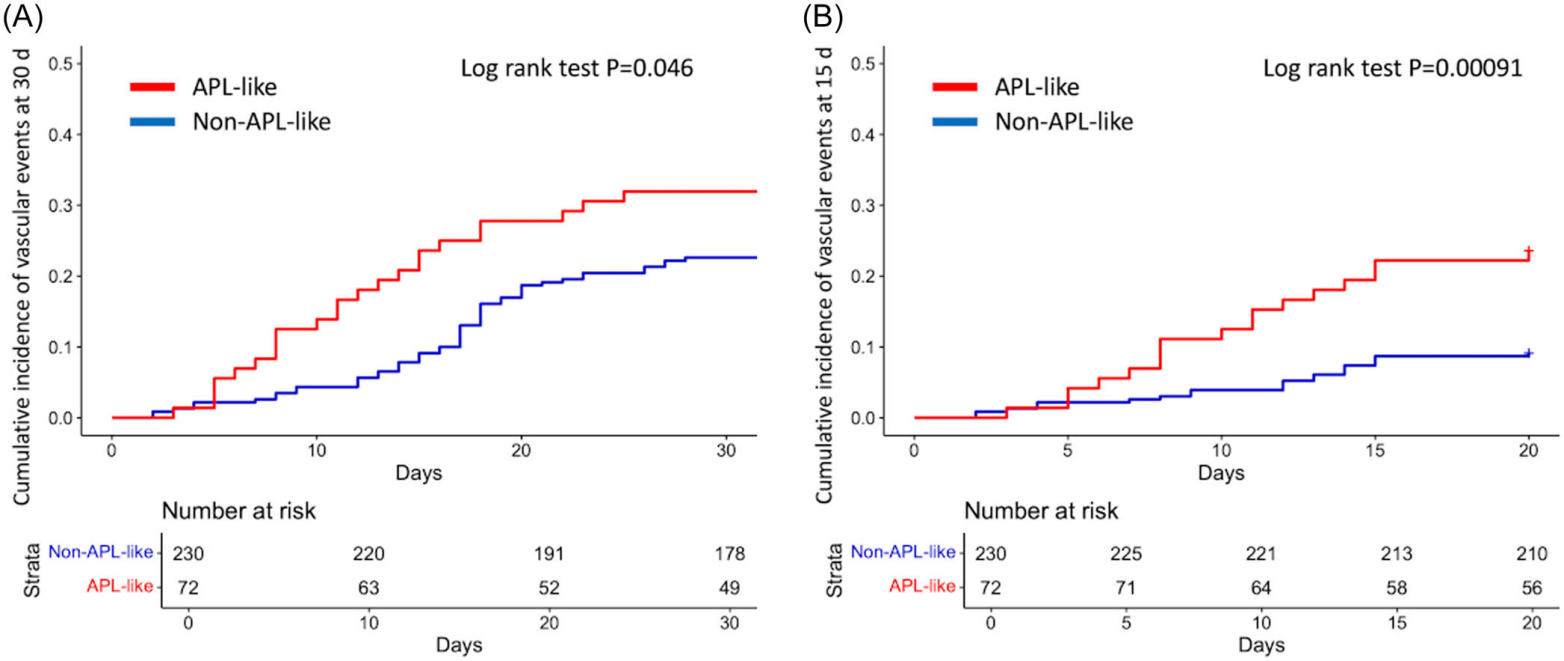
**Cumulative incidence of events according to acute promyelocytic leukemia (APL)‐like signature in the validation cohort.** Plots of cumulative incidence of vascular events at **(A)** 30 days and **(B)** at 15 days. The curves of patients with APL‐like signature are depicted in red; the curves of non‐APL‐like patients are depicted in blue. The curves were created using R software.

Regarding clinically relevant vascular complications, the 30‐day incidence was significantly higher in APL‐like (32.0%) compared to non‐APL‐like patients (22.6%, P = 0.046) (Figure 3A). However, the effect of APL‐like was lost in multivariate analysis (HR = 1.48; P = 0.089), whereas low platelet counts (<50 × 10^9^/L) remained the only significant factor (HR = 0.57; P = 0.017).

As the median time to event occurrence was significantly shorter in APL‐like (12 days) than in non‐APL‐like patients (17.5 days, P = 0.002), we focused our analysis on events occurring within the first 15 days. In this time interval, the difference between APL‐like and non‐APL‐like groups was statistically significant (23.6% vs. 9.9%; P = 0.00091) (Figure 3B) and the independent effect exerted by APL‐like immunophenotype was confirmed by the multivariate analysis (HR = 2.70; 95% CI, 1.4–5.1; P = 0.0025), in addition to leukocytosis (HR = 3.18; 95% CI, 1.7–6.0; P = 0.0004) (Table S8). A landmark analysis of vascular events occurring from Day 15 onwards confirmed the lack of impact of APL‐like immunophenotype on later events (P = 0.42; Figure S6). Similar results were observed when the analysis was restricted to hemorrhages (Figure S7), with a clear effect of APL‐like subset on events occurring within 15 days, which was maintained in multivariate analysis (HR = 2.72; 95% CI, 1.4–5.2; P = 0.002) (Table S9). The higher 15‐day incidence of vascular events in the APL‐like group versus controls was confirmed in both the GO (P = 0.045) and no‐GO (P = 0.0034, Figure S8) treatment arms.

The cumulative incidence of early death at 30 days did not differ significantly between APL‐like (5.6%) and non‐APL‐like patients (4.4%; P = 0.67) (Figure S9). Notably, the early death rate in the validation cohort (3.9%) was lower than in the overall training set (10.2%), but comparable to that observed in intensively treated patients within the training set (3.0%), likely reflecting protocol‐driven patient selection.

Consistent with the results of the training set, the distribution of *NPM1* mutation types did not show any interaction with the APL‐like immunophenotype (Table 2), nor had the mutation type (A vs. non‐A) any effect on vascular events (P = 0.35; Figure S10).

The APL‐like group harbored a significantly higher rate of mutations in *IDH1/2* genes compared to the non‐APL‐like group (58.6% vs. 29.8%, respectively; P < 0.001). Specifically, this difference was mainly due to *IDH2* mutations (35.7% vs. 14.9%, respectively; P = 0.0003), whereas no significant difference was observed for *IDH1* mutations (22.9% vs. 17.5%; P = 0.381). In the validation set, the presence of *IDH1/2* mutations did not show any influence on the incidence of vascular events at 15 (P = 0.18) or 30 (P = 0.5) days (Figure S11). Moreover, APL‐like patients showed a higher incidence of *TET2* mutations (24.3% vs. 12.7%, respectively; P = 0.024). Conversely, *DNMT3A* mutations were confirmed more frequently in the non‐APL‐like compared to the APL‐like subset (65.7% vs. 22.2%, P < 0.001). No significant differences emerged in the frequency of *FLT3* mutations (33.3% vs. 29.1%; P = 0.56) (Table 2).

No significant differences in EFS (HR = 0.86; 95% CI, 0.58–1.27; P = 0.44) or OS (HR = 0.87; 95% CI, 0.55–1.37; P = 0.54) were observed between the APL‐like and non‐APL‐like groups (Figure S12). Then we searched for any interaction between APL‐like immunophenotype and treatment with GO, the subject of randomization in the trial. In the context of the APL‐like group, no differences in EFS (HR = 1.22; 95% CI, 0.61–2.44; P = 0.58) or OS (HR = 1.95; 95% CI, 0.8–4.43; P = 0.11) were observed according to treatment allocation, with the no‐GO arm as the reference group (Figure S13).

## DISCUSSION

The pathogenesis of coagulopathy in patients with newly diagnosed AML is multifactorial, involving the overexpression of tissue factor by blasts, the release of pro‐inflammatory and pro‐coagulant cytokines, platelet activation, and other mechanisms.^24^, ^25^ During the initial management, cytolysis and/or active infections can often contribute to exacerbating the coagulation imbalance, potentially leading to overt DIC.^26^

Although less extensively characterized than in APL, the pathogenesis of coagulopathy in non‐APL AML is generally thought to involve similar biological mechanisms.^27^ However, in current clinical practice, once a diagnosis of APL has been excluded, the management of hemorrhagic and thrombotic risk relies primarily on the clinical picture, complete blood count, and coagulation parameters, but is independent from biological features.^1^

In this study, we provide evidence that the APL‐like immunophenotype is a strong predictive factor for coagulopathy and early vascular events. Patients exhibiting the APL‐like immunophenotype accounted for approximately 25% of *NPM1* ^mut^ cases in our series, were older (median age 66 vs. 58 years, P < 0.001), and had a lower platelet count (40 vs. 57 × 10^9^/L, P = 0.006) compared to other patients. The APL‐like immunophenotype was associated with laboratory abnormalities indicative of coagulopathy, summarized by greater DIC score values, and most importantly with a higher incidence of early vascular events (30.5% vs. 10.4%, P < 0.001, Figure 2A). Of note, the impact of APL‐like immunophenotype on early bleeding events (HR 2.3, P = 0.0061) was independent of age (HR 1.2, P = 0.59), WBC (HR 1.1, P = 0.81), and platelet count (HR 0.60, P = 0.11), suggesting its pathogenesis to be related to the intrinsic characteristics of leukemic blasts. Consistent with the higher occurrence of early vascular (and fatal) manifestations, patients displaying the APL‐like immunophenotype were less likely to receive induction therapy (80.0% vs. 94.4%, P = 0.0002), either intensive or low‐intensity.

Notably, the APL‐like immunophenotype was independently associated with early mortality due to vascular events in our real‐world patient cohort, with a 6.3% of mortality rate compared to 0.35% for the non‐APL‐like (Gray's test P = 0.00015). No differences were seen for other causes of death (P = 0.18).

The analysis of a validation dataset from the AMLSG 09‐09 trial provided additional insights and confirmed the association between the APL‐like immunophenotype and a higher incidence of vascular events at 30 days (P = 0.046, Figure 3A), with even greater significance when censoring events at 15 days from diagnosis (P = 0.0016, Figure 3B). Multivariate analyses showed that the impact of APL‐like immunophenotype was restricted to the first 15‐day period, whereas the occurrence of later events was likely mitigated by the anti‐leukemic treatment, though still influenced by baseline platelet count. We interpret the latter findings as reflecting the exclusion of patients experiencing very early and severe vascular complications from enrollment in a prospective clinical trial. Nonetheless, the fact that events occurring within 15 days from diagnosis remained significantly more frequent in the APL‐like group compared to the counterpart supports the hypothesis of an effect intrinsic to the pathobiological features of the disease. This also likely explains the discrepancy in mortality rate between the real‐world cohort and the AMLSG 09‐09 trial, although an effect related to differences in treatment schedules and a potential influence of ATRA on bleeding/thrombotic events cannot be excluded. Notably, the AMLSG 09‐09 trial included administration of ATRA to all enrolled patients and GO for those randomized to the experimental arm.^13^, ^14^ The higher incidence of vascular events for APL‐like patients was confirmed in the two treatment arms (Figure S8).

The abnormalities in coagulopathy markers observed in association with the APL‐like subset were consistent with previous reports.^28^, ^29^ The main contribution of our study, based on two large series of *NPM1* ^mut^ AML patients, lies in the strong clinical evidence linking the APL‐like immunophenotype to the occurrence of early and potentially fatal vascular events. Our findings clearly ask for the rapid identification and disease‐driven management of this patient category aimed at controlling coagulopathy and minimizing the risk of severe complications.

Although morphological features of blasts were not systematically assessed in our study, they were shown to not ensure the same diagnostic accuracy as in APL.^30^ The description of a “cup‐like” morphology has been associated with *NPM1* mutations in some reports,^28^, ^31^ but it failed to demonstrate sufficient reliability in predicting specific genotypes when evaluated in unselected populations.^32^ Furthermore, this morphological trait has also been described in cases of acute lymphoblastic leukemia.^33^, ^34^

The immunophenotype‐based definition of the APL‐like subset is straightforward, based on a core set of markers routinely assessed in standard diagnostic MFC panels. This allows for rapid turnaround time and fits well with the potential clinical implications. Of note, the consistent proportion of APL‐like cases (approximately 25%) observed both in the training and validation cohorts further supports the reproducibility of its identification.

As in the current approach for APL, defining an APL‐like immunophenotype may justify the adoption of disease‐directed clinical measures, including close laboratory monitoring and stricter transfusion thresholds (i.e., a target platelet count of >30 × 10^9^/L as in APL). The enrichment for *IDH1/2* mutations in this subset, confirmed in both series herein, may suggest a pathological basis for the clinical phenotype related to changes in the transcriptional program of AML cells, potentially inducing a pro‐inflammatory state,^35^ that might contribute to vascular complications.

In perspective, patients presenting with an APL‐like immunophenotype should be promptly evaluated for the presence of these mutations. In analogy to APL, a potential approach to investigate could include the timely adoption of a targeted inhibitor to mitigate the risk of early vascular events, even before the initiation of a full induction treatment, although no clinical data support pre‐emptive targeted therapy yet.

There are some limitations to our analyses. First, as a retrospective study conducted over an extended enrollment period, changes in supportive care practices over time may have influenced early mortality outcomes. Additionally, we observed a higher incidence of vascular events in the non‐APL‐like group within the validation cohort compared to the corresponding control group in the training dataset. This discrepancy may reflect differences in data collection, as the AMLSG 09‐09 trial was a prospective study with more rigorous and systematic reporting of adverse events than typically seen in real‐world settings. Another limitation was the lack of data on ECOG performance status, a variable known to impact early mortality rates,^36^ although not specifically associated with vascular events, as demonstrated for the APL‐like phenotype. The biological basis underlying the increased susceptibility to coagulopathy in the APL‐like subset remains to be elucidated, and we plan to address it within the framework of a prospective multicenter study coordinated by the Gruppo Italiano Malattie EMatologiche dell'Adulto (GIMEMA) cooperative group (RSO number: 3296; GIMEMA AML3025).

In conclusion, we demonstrated in large training and validation datasets of *NPM1* ^mut^ AML patients that the APL‐like immunophenotype, easily defined by flow cytometry using a set of core antigens, was correlated with the presence of coagulopathy and the occurrence of early vascular events, affecting mortality within 30 days from diagnosis. Our results support the need for rapid identification of this AML subtype and the development of studies implementing specific clinical measures to improve patient management and outcome.

## AUTHOR CONTRIBUTIONS

**Francesco Mannelli**: Conceptualization; data curation; writing—original draft. **Francesca Crupi**: Data curation; writing—original draft. **Sara Bencini**: Methodology; data curation. **Michaela Feuring**: Data curation. **Gaia Ciolli**: Data curation. **Matteo Piccini**: Data curation. **Marco Frigeni**: Data curation. **Raffaele Palmieri**: Data curation. **Chiara Sartor**: Data curation. **Barbara Scappini**: Data curation. **Giacomo Gianfaldoni**: Data curation. **Benedetta Peruzzi**: Data curation. **Roberto Caporale**: Data curation. **Antonio Scannella**: Data curation. **Laura Fasano**: Data curation. **Elisa Quinti**: Data curation. **Andrea Pasquini**: Data curation. **Jessica Caroprese**: Data curation. **Leonardo Signori**: Methodology. **Fabiana Pancani**: Methodology. **Chiara Maccari**: Methodology. **Tiziana Ottone**: Data curation. **Daniela Spaeth**: Data curation. **Antonio Curti**: Data curation; writing—review and editing. **Maria Teresa Voso**: Data curation; writing—review and editing. **Francesco Annunziato**: Data curation; writing—review and editing. **Konstanze Döhner**: Data curation; supervision; writing—review and editing. **Adriano Venditti**: Data curation; writing—review and editing. **Francesco Buccisano**: Data curation; writing—review and editing. **Alessandro Rambaldi**: Data curation; writing—review and editing. **Paola Guglielmelli**: Data curation; writing—review and editing. **Hartmut Döhner**: Data curation; supervision; writing—review and editing. **Alessandro M. Vannucchi**: Data curation; supervision; writing—review and editing.

## CONFLICT OF INTEREST STATEMENT

F.M.—speaker bureau: Blueprint, Novartis, AbbVie, Servier, GSK, and Astellas. M.T.V.—speaker bureau: Astellas, Servier, BMS, AbbVie, and Daiichi Sankyo; consultant: Jazz, Servier, and Astellas. A.V.—research funding: Jazz Pharmaceuticals; consultancy: Amgen, Servier, AstraZeneca, Pfizer, Kyte‐Gilead, AbbVie, Janssen, Astellas, Astex, Otzuka, Stemline Menarini, BMS, Glycostem, Novartis, and Delbert; all are not related to this manuscript. H.D.—consultancy with honoraria: AbbVie, Otsuka, Pfizer, Servier, and Syndax; clinical research funding (institution): AbbVie, Astellas, Bristol Myers Squibb, Jazz Pharmaceuticals, and Servier; travel, accommodations, expenses: AbbVie and Servier. A.M.V.—honoraria: Novartis, Incyte, GSK, Blueprint, AbbVie, Italfarmaco, and AOP. F.C., S.B., M.F., G.C., M.P., R.P., C.S., B.S., G.G., B.P., R.C., A.S., L.S., E.Q., A.P., J.C., L.S., F.P., C.M., D.S., A.C., F.B., A.R., F.A., K.D., and P.G. declare no competing financial interests.

## ETHICS STATEMENT

The study was approved by the local institutional review board (IRB: project MYNERVA, approval 14560). Written informed consent was obtained from study patients in accordance with the Declaration of Helsinki.

## FUNDING

The work was supported by the AIRC 5×1000 call “Metastatic disease: the key unmet need in oncology” to the MYNERVA project, #21267 (MYeloid NEoplasms Research Venture AIRC). The study was also supported by Legato Zottola to Dipartimento di Medicina Sperimentale e Clinica, Università di Firenze.

## Supporting information

Supporting Information.

## Data Availability

The data that support the findings of this study are available on request from the corresponding author. The data are not publicly available due to privacy or ethical restrictions.
