# Supplementary material for: APL‐like subset within NPM1‐mutated AML: A distinct immunophenotype correlating with early vascular complications
Source: Hemasphere. 2026 Apr 16;10(4):e70307. doi: 10.1002/hem3.70307 (PMC13086621; doi:10.1002/hem3.70307)
Supplement: Supplementary file 1 — Supporting Information. [file HEM3-10-e70307-s001.docx]

Supplemental data

**APL-like subset within *NPM1*-mutated acute myeloid leukemia: a distinct phenotypic signature correlating with early vascular complications**

**A. Supplemental Materials and Methods (pp 2-4)**

S1. Treatment protocols

S2. Multiparametric flow cytometry (MFC) methods for immune-phenotypic characterization at AML diagnosis

S3. Molecular genetics

**B. Supplemental Figures and Legends (pp 5-17)**

S1. APL-like immunophenotypic signature: illustrative example

S2. Cumulative incidence of vascular events according to *NPM1* mutation type

S3. Cumulative incidence of death at 30 days.

S4. Cumulative incidence of vascular events according to *IDH1/2* mutation status

S5. Cumulative incidence of vascular events in intensively treated pts (training cohort)

S6. Landmark analysis of incidence of vascular events from day 15 onwards in AMLSG 09-09 trial

S7-S11. Cumulative incidence of hemorrhagic events and death in AMLSG 09-09 trial according to *NPM1* mutation type, APL-like phenotype, *IDH1/2* mutation status and treatment arm

S12. Event-free and overall survival in AMLSG 09-09 clinical trial according to APL-like phenotype.

S13. Event-free and overall survival in patients with APL-like phenotype enrolled in AMLSG 09-09 clinical trial according to treatment arm.

**C. Supplemental Tables (pp 18-27)**

S1-S3. Multivariate Analyses in the training cohort.

S4. Details of fatal vascular events occurring in the training cohort.

S5-S6. Propensity score matching analysis.

S7. Characteristics of patients in learning and validation cohorts.

S8-S9. Multivariate Analyses in the validation cohort.

**Supplemental Materials and Methods**

**S1. Treatment protocols**

Protocol-1: since April 2004 to March 2007, patients received induction according to standard-dose cytarabine (SDAC) based course, namely “3+7” (Cytarabine 100 mg/sqm bid on days 1-7; Idarubicin 12 mg/sqm on days 1-3). From 2006 on, etoposide 100 mg/sqm on days 1-5 was added (ICE course). High-dose cytarabine (HDAC) 1, 3, 5 (3000 mg/sqm bid on days 1, 3, 5) was used as first consolidation in patients aged < 61 years attaining complete remission (CR) after ICE. Patients with persistent disease (*i.e.,* > 5% BM blasts at hematopoietic recovery) after first course received a salvage regimen (Ida-HDAC). In an intention-to-treat approach, patients aged < 55 years with high-risk karyotype, *FLT3*-ITD or adverse clinical features (secondary AML, CR after second course, hyperleukocytosis) were assigned to undergo allogeneic stem cell transplantation (SCT) from matched related or unrelated donor. Patients with intermediate cytogenetic risk in the absence of *FLT3*-ITD and adverse clinical features were allocated to allogeneic SCT if a related donor was available. Autologous SCT was offered to patients aged < 61 y with low-risk cytogenetics, intermediate-risk cytogenetics without sibling donor and high-risk disease not eligible to allogeneic SCT. Peripheral blood (PB) stem cells for autologous SCT were collected after a mobilization course (Cytarabine 500 mg/sqm bid on days 1-6; Daunorubicin 50 mg/sqm on days 4-6). Patients who failed mobilization received two additional courses with high dose cytarabine.

Protocol-2: since April 2007 to April 2014, patients were treated according to Northern Italy Leukemia Group (NILG) AML 02-06 protocol. Until March 2012, patients were recruited within the NILG AML 02/06 trial [(ClinicalTrials.gov Identifier: NCT00495287; reference: Bassan R, et al; Blood Adv. 2019;3(7):1103–1117)]. From April 2012, after closure of NILG AML 02/06 trial, patients were treated according to the standard arm provided by the protocol. The protocol provided a randomization at induction between a standard ICE induction *versus* an experimental intensified one. Patients aged > 65 y were treated according to standard arm. Upon CR achievement, patients received standard doses cytarabine consolidation and were divided into standard and high-risk cases (SR, HR): SR: favorable or intermediate risk cytogenetics (according to SWOG criteria) without any adverse clinical factor (secondary AML, *FLT3*-ITD, CR after cycle 2, persistence of pre-existing cytogenetic abnormality despite morphological CR; total WBC count >50 x10^9^/L); HR: all non-SR cases. HR patients were assigned to undergo allogeneic SCT. Provided sufficient CD34+ cells were previously collected (>2x10^6^/kg) upon recovery from high doses cytarabine, SR patients and HR patients excluded from allo-SCT and aged 65 years or less were randomized between autologous SCT and high doses consolidation therapy (R2). HR/SR patients unable to be randomized in R2 because of inadequate blood stem cell yield received intermediate-dose consolidation. Patients randomized to experimental arm were excluded from outcome analysis.

Protocol-3: since May 2014 to April 2017, patients received induction according to Ida-FLA course, (Cytarabine 2000 mg/sqm on days 1-4; Fludarabine 30 mg/sqm on days 1-4; Idarubicin 10 mg/sqm on days 2-4). High-dose Cytarabine (3000 mg/sqm bid days 1, 3, 5) was used as first consolidation in patients aged < 61 years attaining complete remission (CR). Patients with persistent disease (*i.e.* > 5% BM blasts at hematopoietic recovery) after first course received a salvage regimen (Clofarabine-based). In post CR phase, patients were stratified according to European Leukemia Net 2010 guidelines [reference: Döhner H, et al; Blood. 2010;115(3):453–474]. Patients in adverse-risk category were allocated to allogeneic HSCT from matched related or unrelated donor. Patients in intermediate category were allocated to allogeneic SCT if a related donor was available. Patients in favorable-risk ELN category and high-risk disease not eligible to allogeneic SCT received up to two additional courses with high dose cytarabine.

Protocol-4: since 2017, patients harboring *FLT3* mutations received induction according to “3+7” scheme (Cytarabine 200 mg/sqm intravenous continuous infusion on days 1-7; Daunorubicin 60 mg/sqm on days 1-3) + Midostaurin 50 mg bid orally on days 8-21. High-dose Cytarabine (3000 mg/sqm bid days 1, 3, 5) + Midostaurin 50 mg bid orally on days 8-21 was used as first consolidation in patients aged < 61 years attaining complete remission (CR) [reference: Stone R, New Engl J Med. 2017;377, 454]. In post CR phase, patients were stratified according to European Leukemia Net 2017 guidelines [reference: Döhner H, et al; Blood. 2022;140 (12): 1345]. Patients in adverse-risk category were allocated to allogeneic HSCT from matched related or unrelated donor. Patients in intermediate category were allocated to allogeneic SCT if a related donor was available. Patients in favorable-risk ELN category and high-risk disease not eligible to allogeneic SCT received up to two additional courses with high dose cytarabine.

Protocol-5: since 2017, elderly patients (>60 y) diagnosed with AML with myelodysplasia-related changes received induction with CPX-351 100 U/sqm intravenously on days 1, 3, 5. For patients in CR after induction, consolidation treatment provided up to two cycles of CPX-351 65 U/sqm intravenously on days 1, 3 [reference: Lancet J, et al; JCO. 2016; 36: 2684]. If eligible, patients were allocated to allogeneic HSCT from matched related or unrelated donor.

Protocol-6: since 2017, patients diagnosed with core binding factor (CBF) related AML received induction according to “3+7” scheme (Cytarabine 200 mg/sqm intravenous continuous infusion on days 1-7; Daunorubicin 60 mg/sqm on days 1-3) + Gemtuzumab Ozogamicin intravenously 3 mg/m² (dose capping at 5 mg] on days 1, 4, and 7. Patients in CR received two consolidation courses of intravenous daunorubicin (60 mg/m² for 1 day or 2 days) in combination with intravenous ARA-C (1000 mg/sqm iv bid on days 1–4) + Gemtuzumab Ozogamicin 3 mg/m² (dose capping at 5 mg] on days 1 [reference: Castaigne S, et al; Lancet. 2012; 379: 1508]. Patients with CBF-related AML were not allocated to allogeneic HSCT in first CR.

**S2. Multiparametric flow cytometry (MFC) methods for immune-phenotypic characterization at AML diagnosis**

The immunophenotypic profile at AML diagnosis were assessed using multiple combinations including CD45 conjugated with peridinin chlorophyll protein (PerCP or PerCP-Cy5.5). The panel of diagnostic monoclonal antibodies (MoAb) was previously established and reported elsewhere^a^. A FACSCanto II flow cytometer (Becton Dickinson, BD, San Jose, CA) was used equipped with FACSDiva Software (BD) for data acquisition. Instrument setup, calibration and quality control were performed to ensure measures’ stability^b^. Consistency of fluorescence intensity was monitored weekly by running fluorochrome-conjugated beads (CS&T, BD). Fluorescence photomultiplier voltages were adjusted until the mean channel values for the unlabelled beads corresponded to predetermined target values. Overtime stability of bead mean fluorescence intensity (MFI) profile was checked by Levey-Jennings diagrams; changes of up to ±15% of the mean target MFI were tolerated. The mixed-bead suspension was used to determine the appropriate compensation settings. Each combination of MoAbs was added to 50 μl of a suspension of BM cells adjusted to 20,000 nucleated cells/μl; a stain-lyse-and-then-wash procedure was adopted.

**References**

1. Mannelli F, Gianfaldoni G, Bencini S, et al. Early peripheral blast cell clearance predicts minimal residual disease status and refines disease prognosis in acute myeloid leukemia. Am J Hematol 2020;95(11):1304–1313.
2. Owens MA, Vall HG, Hurley AA, Wormsley SB. Validation and quality control of immunophenotyping in clinical flow cytometry. Journal of Immunological Methods. 2000;243(1–2):33–50.

**S3. Molecular Genetics:**

*NPM1 mutations*: *NPM1*_mut_ AML was defined by immunohistochemical criteria and/or by mutational analysis, as specified below. Total RNA was extracted from Ficoll–Hypaque isolated mononuclear cells. RNA was reverse transcribed using random examers. *NPM1* exon 12 was amplified as previously described with minor modification (*Noguera et al, Leukemia 2005;19:1479–82*). Briefly, 2 µl of cDNA were amplified in a total volume of 25 µl of the reaction mixture containing 1x PCR buffer, 0.2mM dNTP, 2mM MgCl2, 1.25 U Hot Start Taq polymerase, and 10 pmol of each primer (HEX NPM-Rev6 5’- ACCATTTCCATGTCTGAGCACC-3’, NPM-F25’-ATCAATTATGTGAAGAATTGCTTAC-3’). Pre-heating of the mixture at 94°C for 5 min was followed by 30 cycles of 30 s at 94°C, 45 s at 57°C, and 45 s at 72°C. A final extension of 10 min was carried out at 72°C on a Gene Amp PCR System 2400 (Perkin Elmer, Emeryville, CA, USA). NPM1 amplified product was separated with a capillary electrophoresis-based system (ABI PRISM 310 genetic analyzer, Applied Biosystems).

*FLT3*-ITD mutation: Approximately 100 ng of genomic DNA was amplified to produce a 328 bp fragment from wild-type alleles. Amplification was performed for 30 cycles (30 seconds at 95°C, 30 seconds at 60°C, 30 seconds at 72°C) followed by 40 minutes at 72°C, in a reaction mix containing 1x buffer, 2.0 mM MgCl2, 0.2mM dNTPs and 10 pmol each primer (11F: FAM 5’-GCAATTTAGGTATGAAAGCCAGC-3’ and 12R: 5’-CTTTCAGCATTTTGACGGCAACC-3’) and 1.25U Hot Start Taq polymerase in a total volume of 25 uL. FLT-3 amplified product was separated with a capillary electrophoresis-based system (ABI PRISM 310 genetic analyzer, Applied Biosystems).

*CEBPA* mutations: Mutations of *CEBPA* gene were detected by genomic DNA PCR and direct sequencing. The primer sets are those designed by Pabst et al (*Nat Genet. 2001;27:263*). Briefly, three overlapping primer pairs were used to amplify the entire coding region of human *CEBPA*: *CEBPA* AF-TCGCCATGCCGGGAGAACTCTAAC, CEBPA AR-AGCTGCTTGGCTTCATCCTCCT (548bp); *CEBPA* BF-CCGCTGGTGATCAAGCAGGA, CEBPA BR-CCGGTACTCGTTGCTGTTCT (390bp); *CEBPA* CF-CAAGGCCAAGAAGTCGGTGGACA, CEBPA CR-CACGGTCTGGGCAAGCCTCGAGAT (356bp). PCR reactions were run in a final volume of 50 µL containing genomic DNA (300 ng), KCl (50 mmol/L), Tris-HCl (20 mmol/L, pH 8.4), MgCl2 (2.5 mmol/L), 5% volume DMSO, primers (2 mmol/L of each), nucleotides (0.1 mmol/L of each), and Taq DNA polymerase (1U). PCR conditions were 94°C for 45 seconds, 62°C for 45 seconds and 72°C for 45 seconds for 45 cycles, with a final step for 10 minutes at 72°C. PCR products were sequenced using BigDye Terminator Cycle Sequencing Kit o v1.1 kit (Applied Biosystems) on ABI 3730 Genetic Analyzer (Applied Biosystems).

**Supplemental Figures and Legends**

**Figure S1.** APL-like immunophenotypic signature: illustrative example. Bivariate dot plots display the antigen pattern for key antigens in a typical case of APL-like *NPM1*^mut^ AML. Blast cells (red) were identified based on dim reactivity for CD45 and low/intermediate side scatter (panel A) and characterized as expressing CD117 and CD33 with negativity for CD34, HLA-DR and CD13 (panels B-D). Dot plots were created with Infinicyt software.


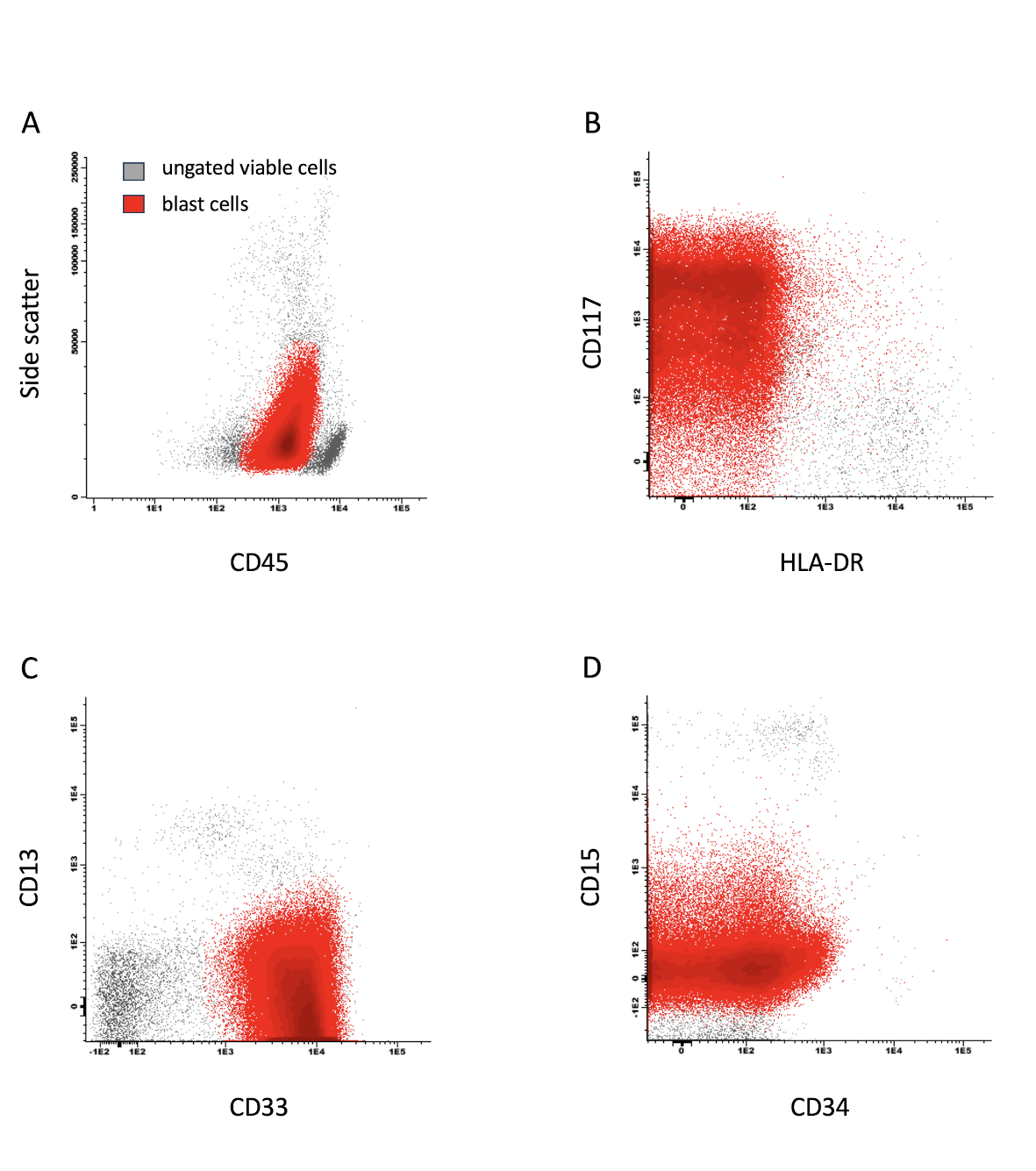


**Figure S2.** Analysis of cumulative incidence of vascular events at 30 days according to *NPM1* mutation type in the training cohort.

**
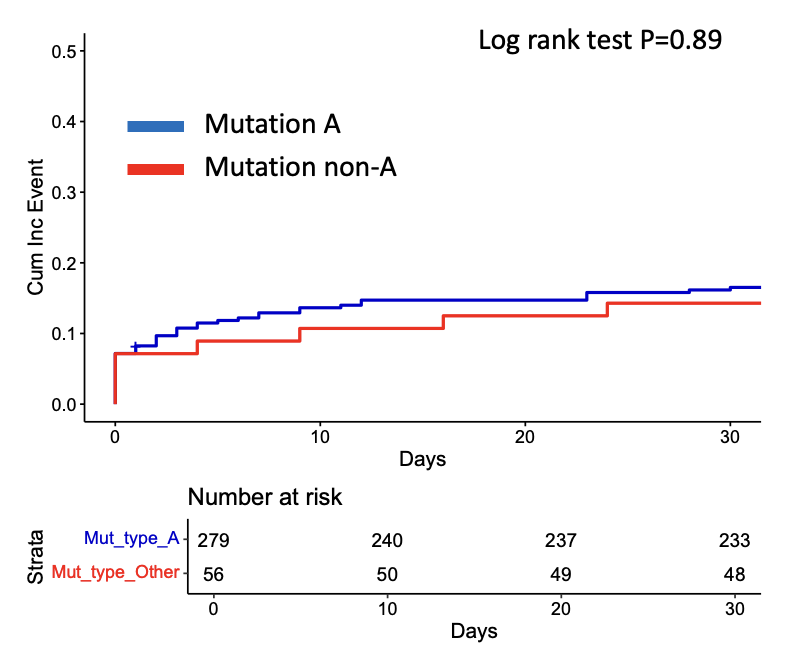
**

**Figure S3.** Analysis of cumulative incidence of death at 30 days according to causes (vascular vs other).

**
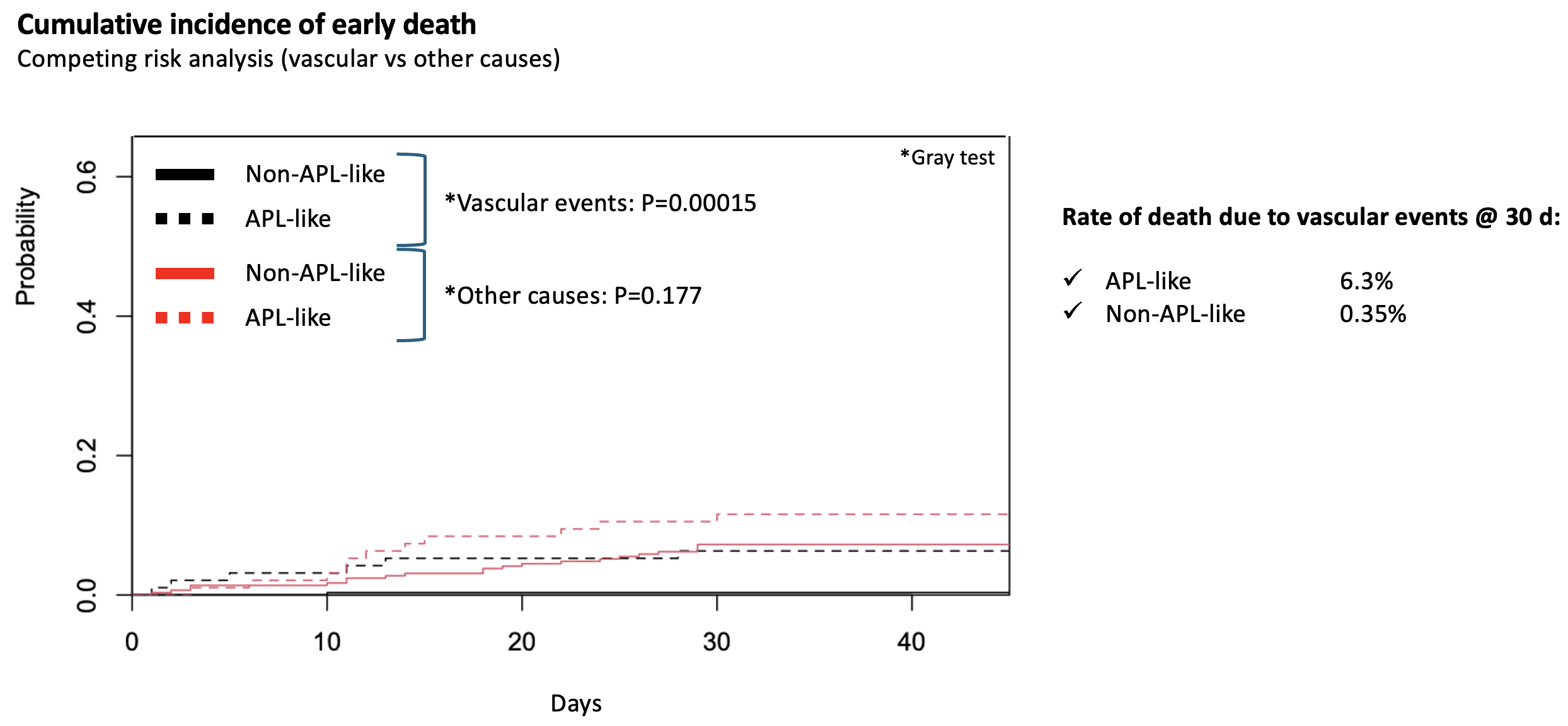
**

**Figure S4.** Cumulative incidence of vascular events according to (A) *IDH1*, (B) *IDH2*, (C) *IDH1/2* mutation status.

**
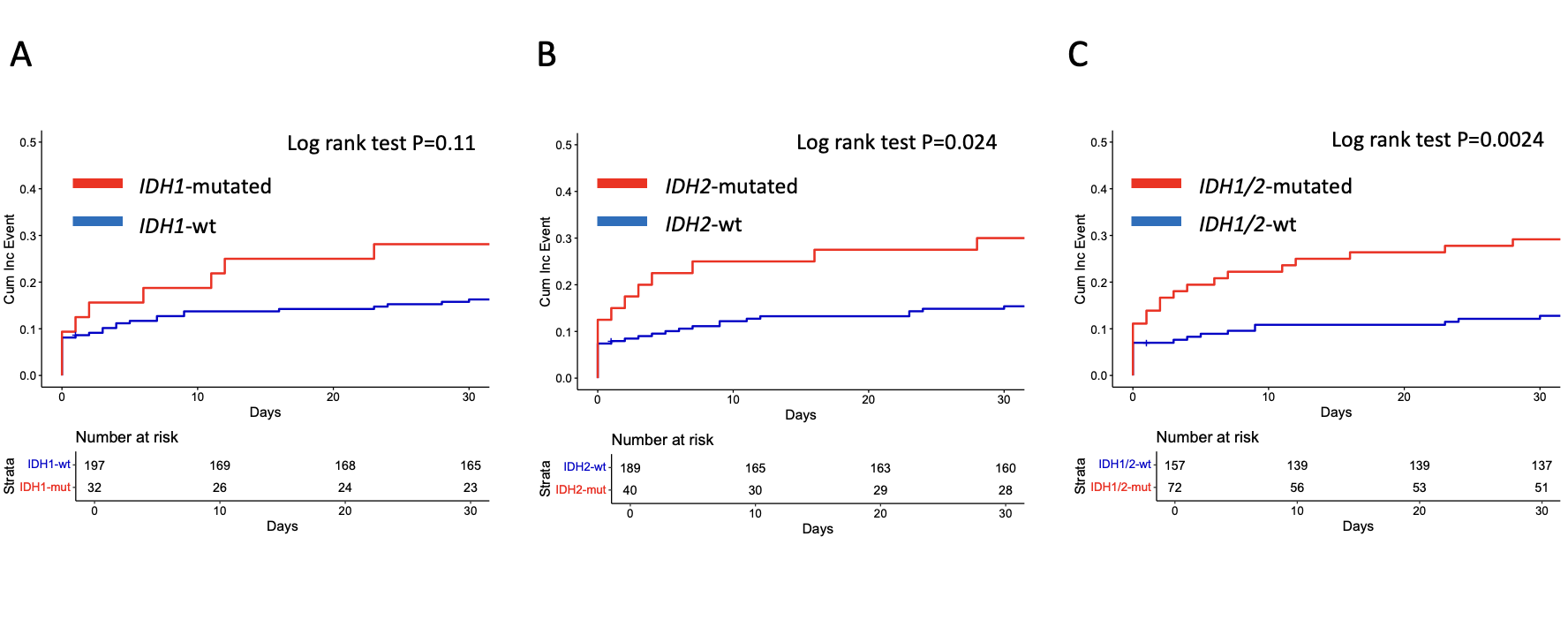
**

**Figure S5.** Cumulative incidence of vascular events in intensively treated pts within the training cohort.

**
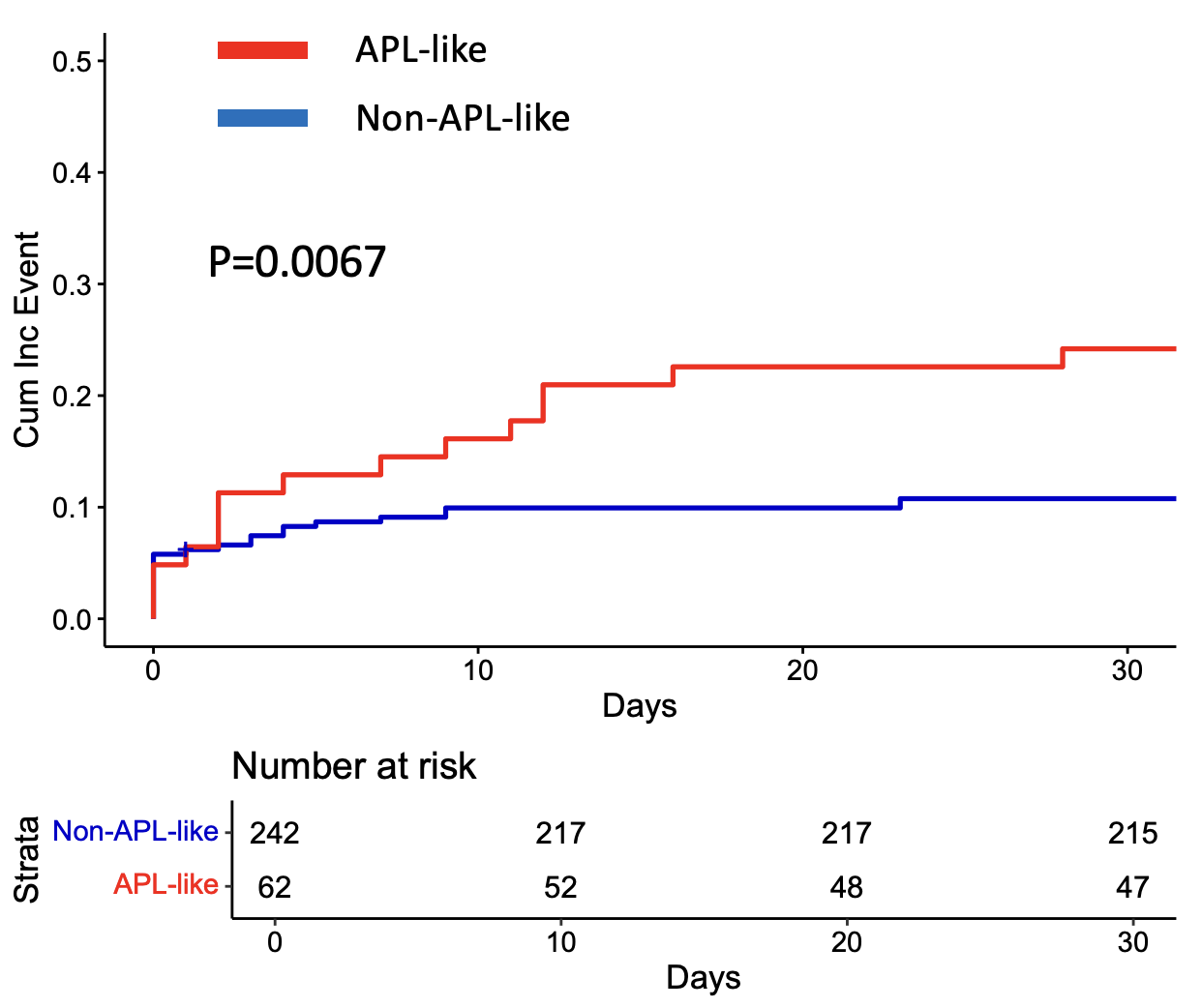
**

**Figure S6.** Landmark analysis of incidence of vascular events from day 15 onwards in AMLSG 09-09 clinical trial according to APL-like phenotype.

**
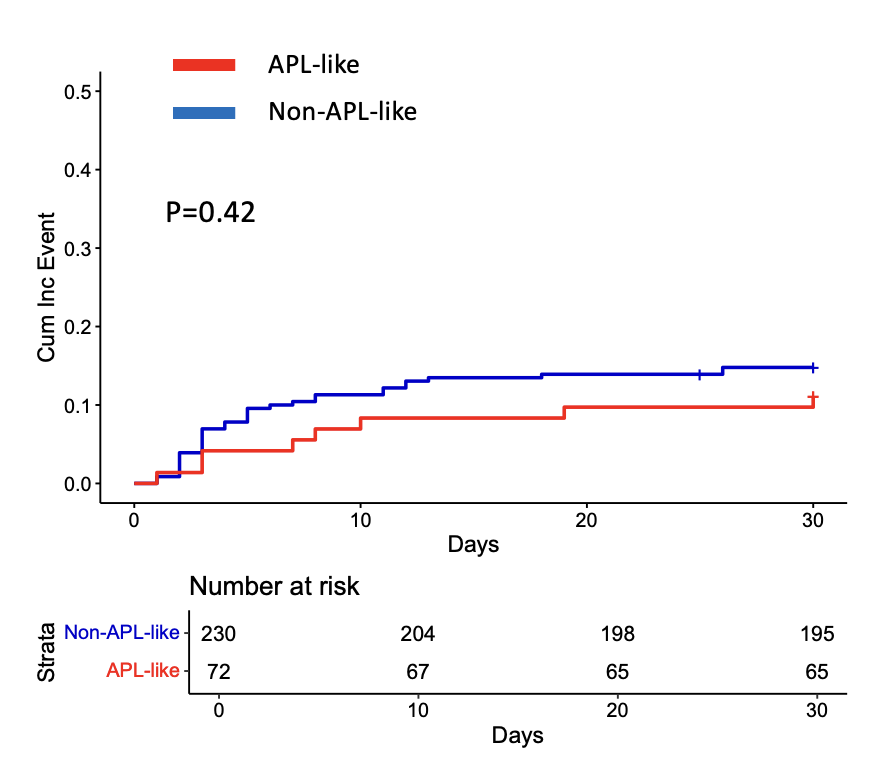
**

**Figure S7.** Cumulative incidence of hemorrhagic events at 30 days (A) and 15 days (B) in AMLSG 09-09 clinical trial according to APL-like phenotype.

**
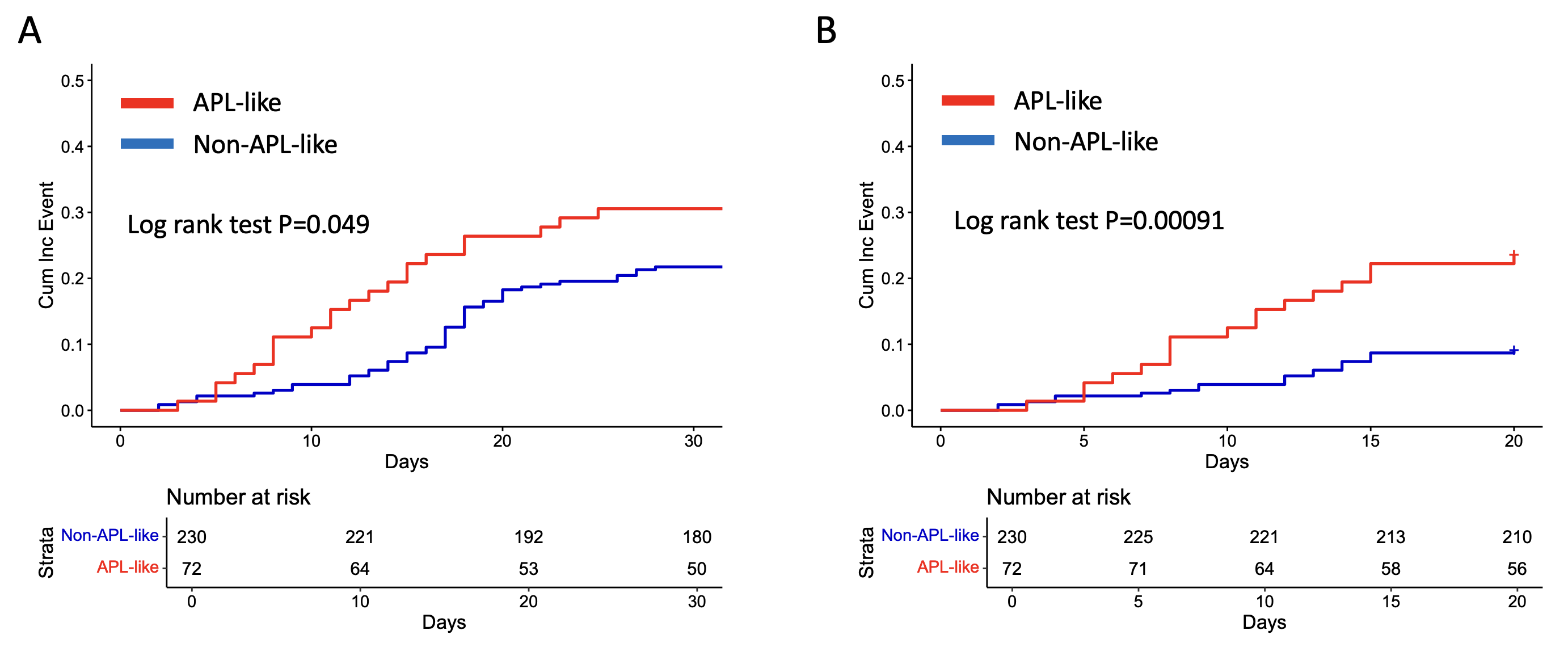
**

**Figure S8.** Cumulative incidence of vascular events at 15 days in the validation cohort according to treatment arm: (A) without gemtuzumab ozogamicin; (B) with gemtuzumab ozogamicin.

**
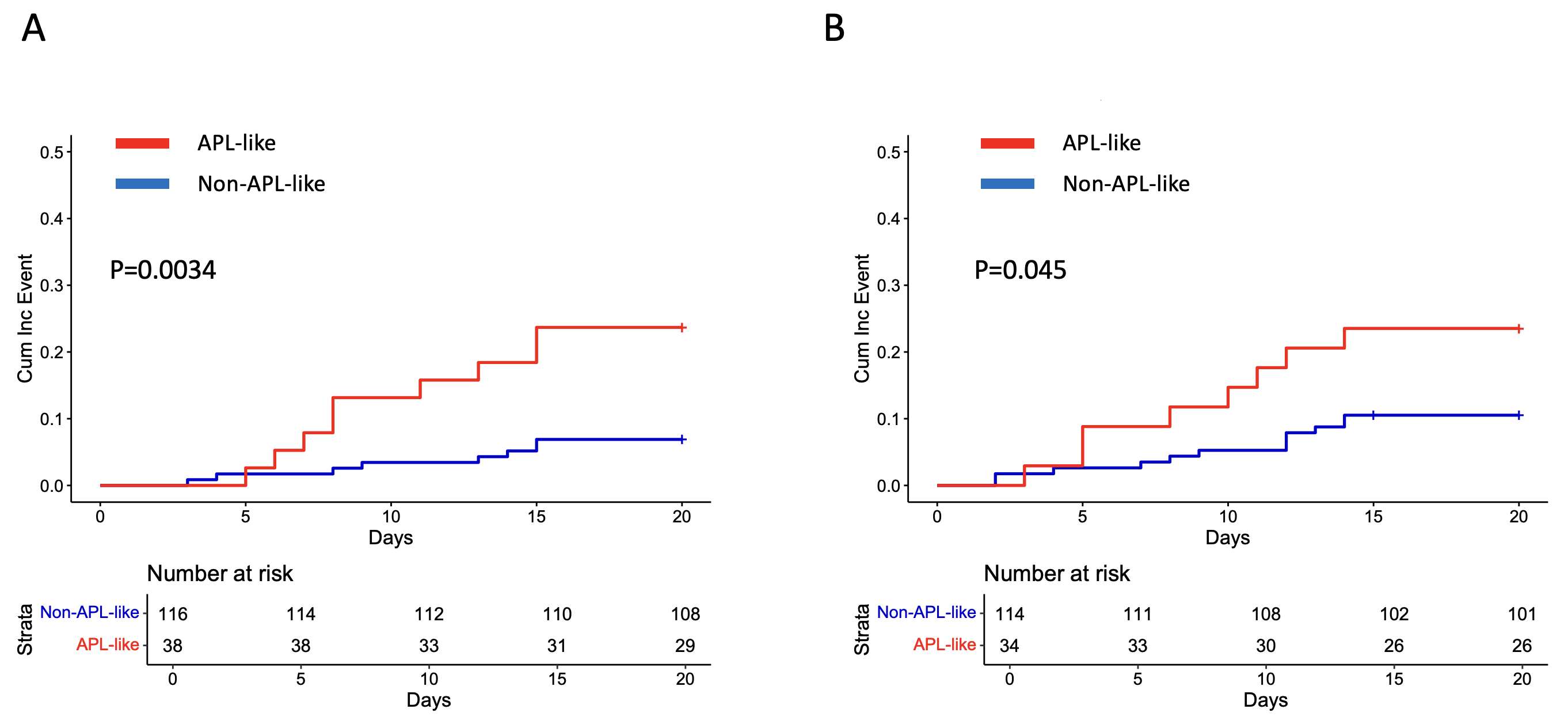
**

**Figure S9.** Cumulative incidence of death within 30 days in AMLSG 09-09 clinical trial according to APL-like phenotype.

**
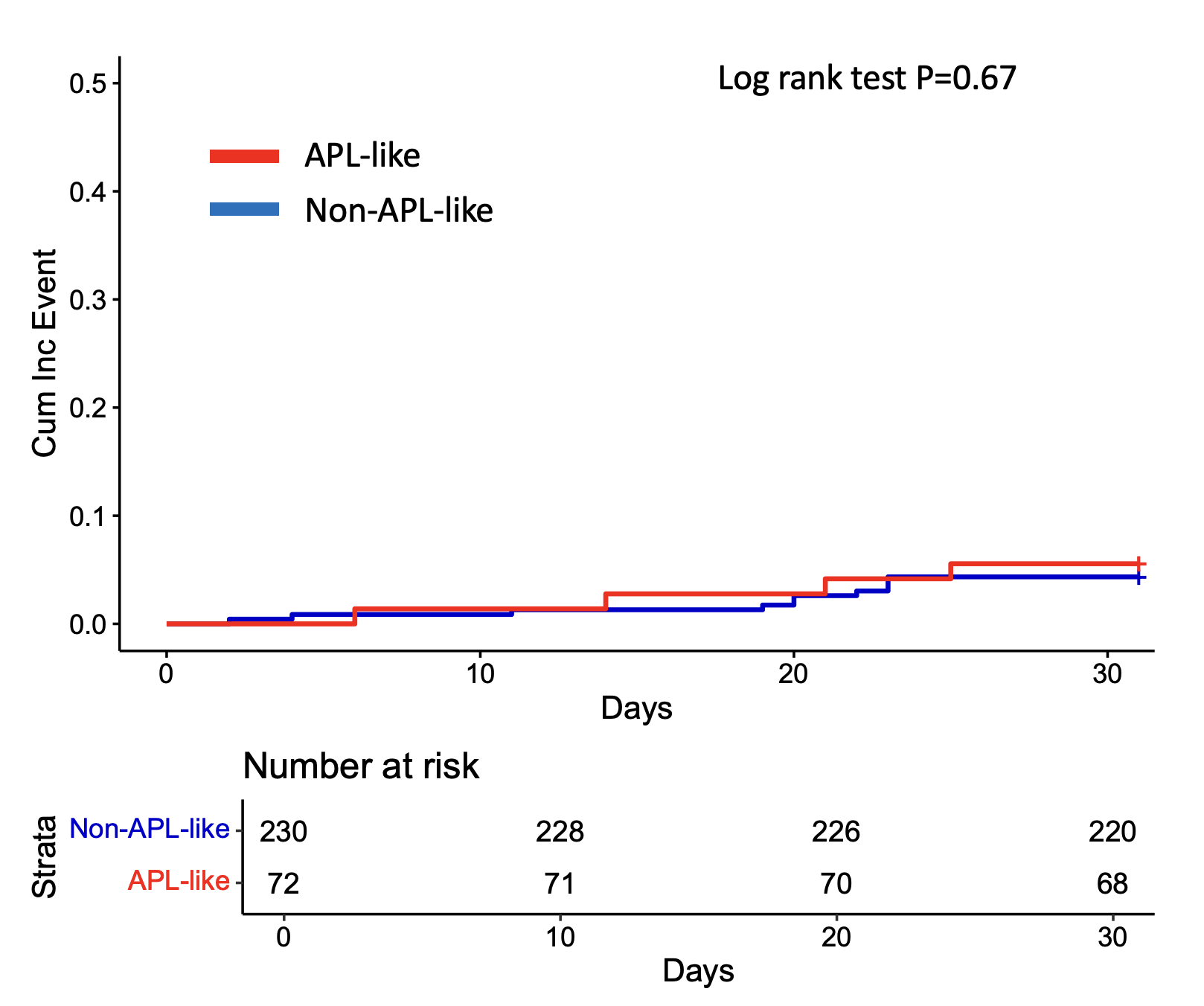
**

**Figure S10.** Cumulative incidence of vascular events within 30 days in AMLSG 09-09 clinical trial according to *NPM1* mutation type.

**
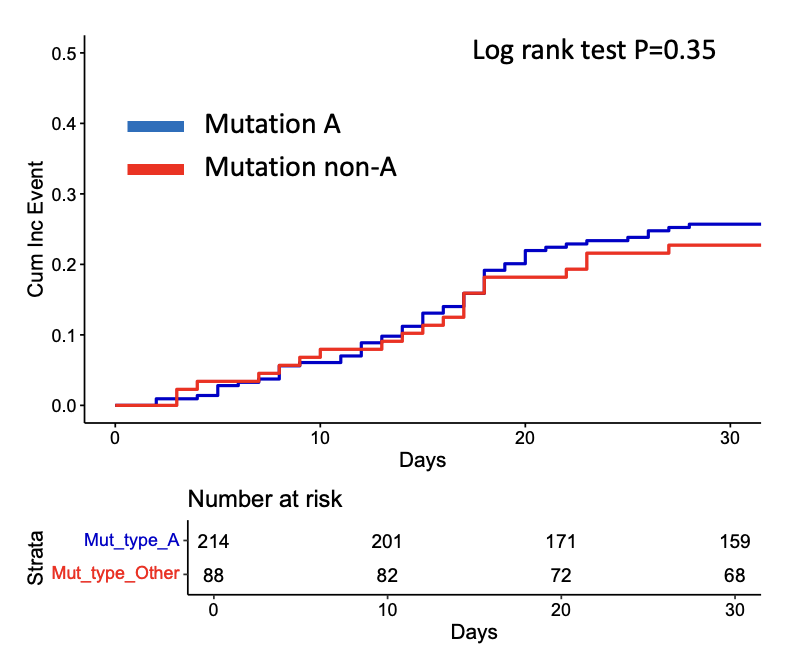
**

**Figure S11.** Cumulative incidence of vascular events within 30 days in AMLSG 09-09 clinical trial according to *IDH1/2* mutation status at 15 days (A) and at 30 days (B).

**
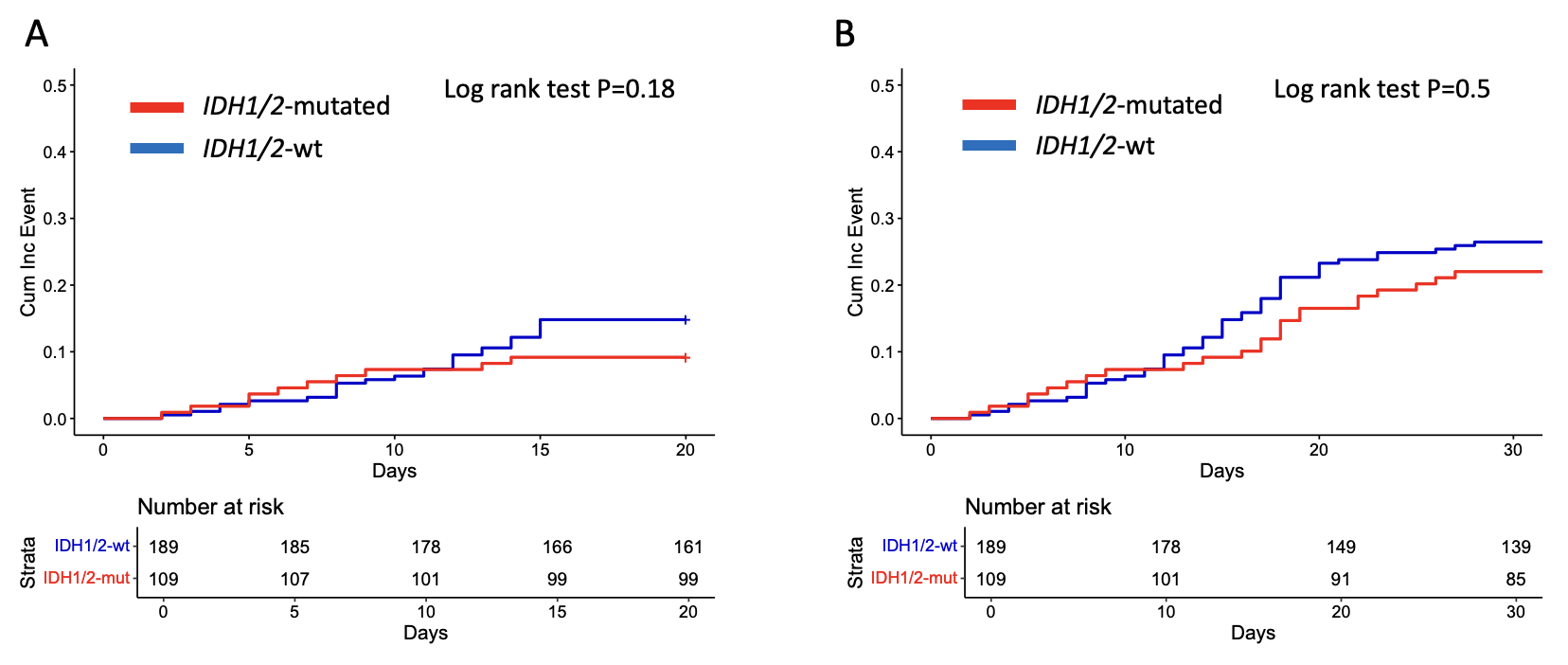
**

**Figure S12.** Event-free (A) and overall (B) survival in AMLSG 09-09 clinical trial according to APL-like phenotype.

**
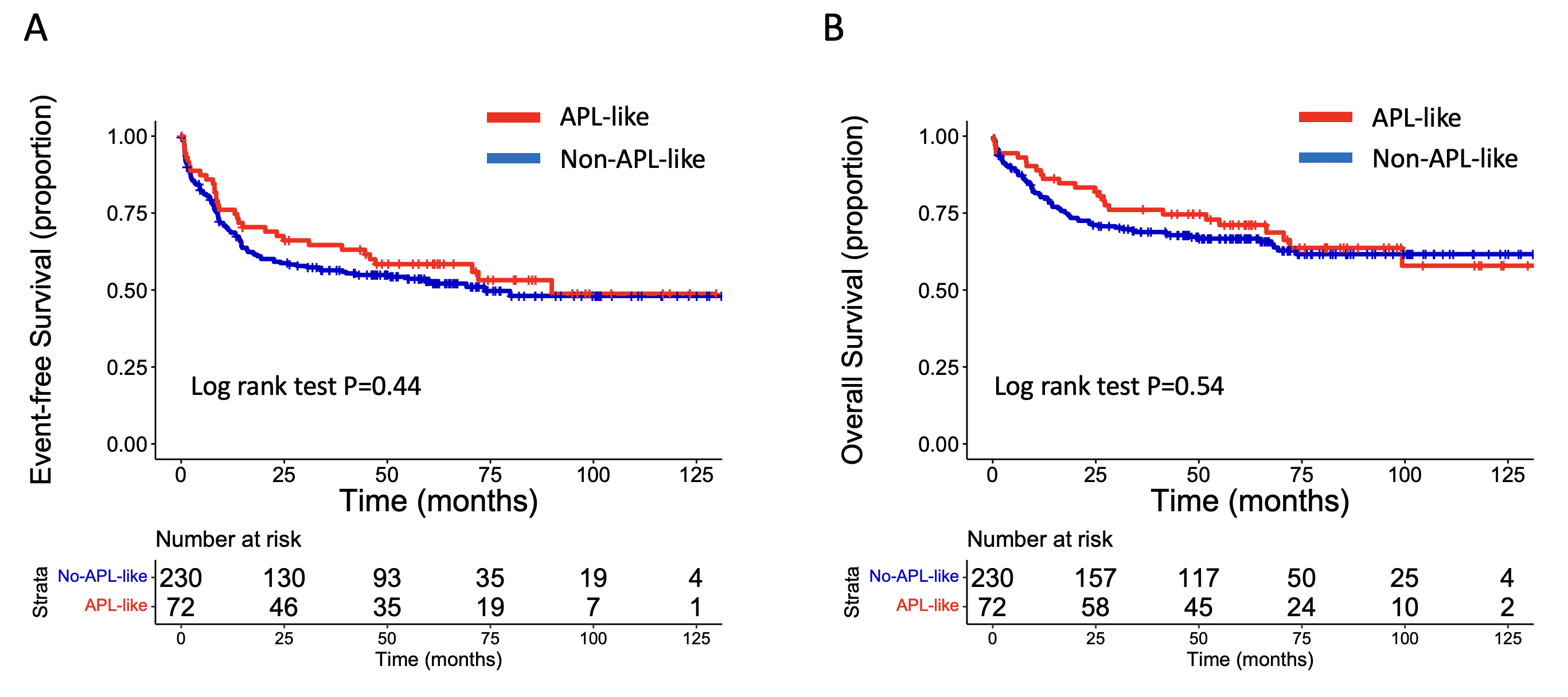
**

**Figure S13.** Event-free (A) and overall (B) survival in patients with APL-like phenotype enrolled in AMLSG 09-09 clinical trial according to treatment arm.

**
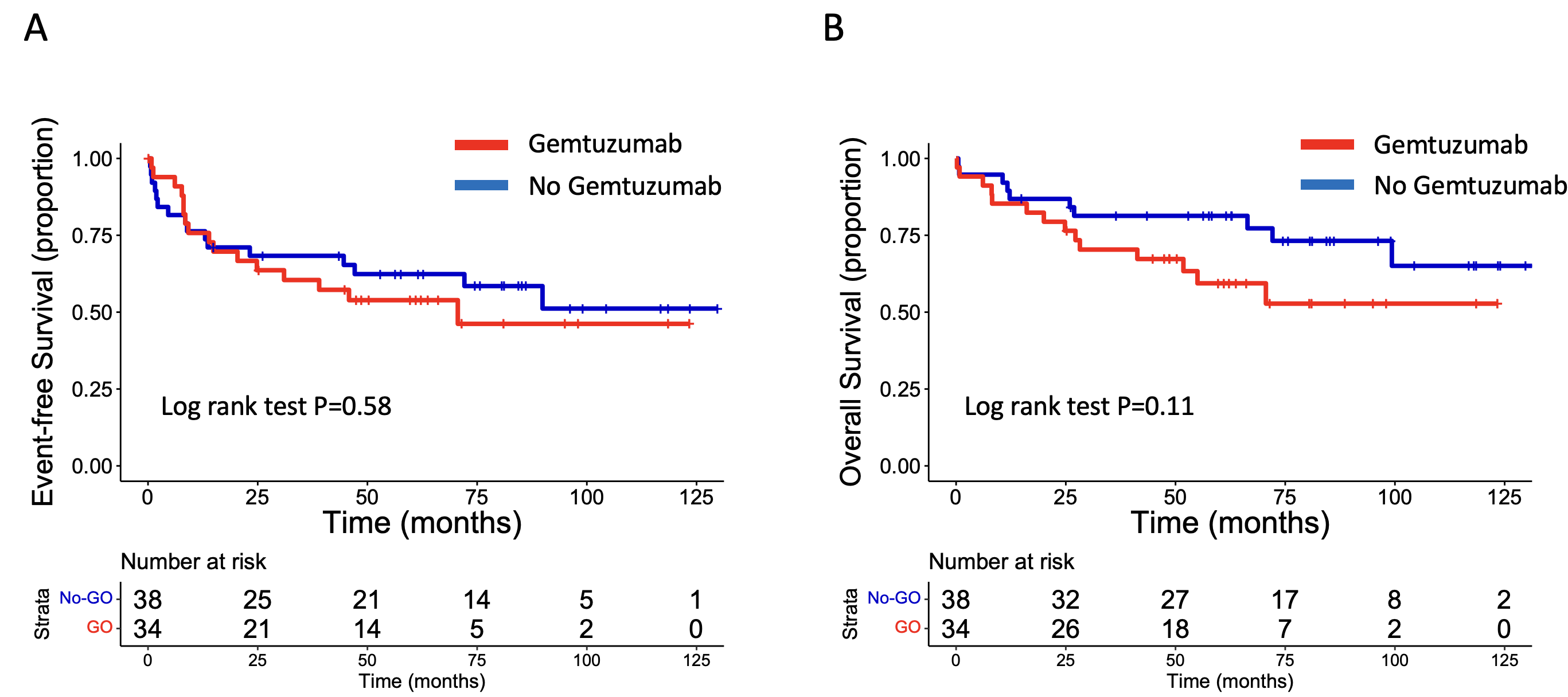
**

**Supplemental Tables and Results**

**Table S1.** Multivariate Analysis (Backward stepwise Cox Regression) for vascular events at 30 days in the training cohort.

|  | **Covariates** | **HR (95% CI)** | **p value** |
| --- | --- | --- | --- |
| **Vascular events at 30 days** | Age (</≥ 60 y) | 1.1 | 0.73 |
|  | WBC (</≥ 50x10^9^/L) | 1.9 (1.1-3.3) | **0.011** |
|  | Plt count (</> 50× 10⁹/L) | 0.76 | 0.35 |
|  | APL-like phenotype | 3.0 (1.8.4.9) | **<0.001** |
| **Hemorrhagic events at 30 days** | Age (</≥ 60 y) | 1.2 | 0.59 |
|  | WBC (</≥ 50x10^9^/L) | 1.1 | 0.81 |
|  | Plt count (</> 50× 10⁹/L) | 0.6 | 0.11 |
|  | APL-like phenotype | 2.3 (1.3.4.1) | **0.0061** |

**Table S2.** Multivariate Analysis (Backward stepwise Cox Regression) for vascular events at 30 days in the training cohort.

|  | **Covariates** | **HR (95% CI)** | **p value** |
| --- | --- | --- | --- |
| **Vascular events at 30 days** | INR (</≥ 1.5) | 1.45 | 0.47 |
|  | Fibrinogen (</≥ 150 mg/dl) | 2.5 | 0.07 |
|  | DIC score (</> 4) | 2.69 (1.1-6.4) | **0.024** |
|  | Plt count (</> 50× 10⁹/L) | 1.05 | 0.90 |
|  | APL-like phenotype | 2.24 (1.1-4.4) | **0.017** |

**Table S3.** Multivariate Analysis (Backward stepwise Cox Regression) for mortality at 30 days in the training cohort.

|  | **Covariates** | **HR (95% CI)** | **p value** |
| --- | --- | --- | --- |
| **Death at 30 days** | Age (</≥ 60 y) | 5.8 (2.4-14.0) | **0.0001** |
|  | WBC (</≥ 50x10^9^/L) | 2.0 (1.1-3.8) | **0.03** |
|  | Plt count (</> 50× 10⁹/L) | 0.35 (0.17-0.68) | **0.002** |
|  | APL-like phenotype | 1.5 | 0.26 |
| **Death due to vascular event at 30 days** | Age (</≥ 60 y) | 1.5 | 0.61 |
|  | WBC (</≥ 50x10^9^/L) | 2.6 | 0.26 |
|  | Plt count (</> 50× 10⁹/L) | 0.26 | 0.16 |
|  | APL-like phenotype | 19.0 (2.3-158) | **0.0063** |

**Table S4**. Details of fatal vascular events occurring in the training cohort.

| **Description of events** | **Days (from diagnosis)** | **Age** | **Phenotype** | **WBC x (10^9^/L)** | **Plt (x10^9^/L)** | **Start of treatment (Yes/No)** |
| --- | --- | --- | --- | --- | --- | --- |
| Intracranial hemorrhage | 7 | 60 | Non-APL-like | 22 | 49 | Yes |
| Intracranial hemorrhage | 6 | 63 | APL-like | 27.5 | 66 | No |
| Intracranial hemorrhage | 13 | 66 | APL-like | 60.1 | 40 | No |
| Intracranial hemorrhage | 1 | 78 | APL-like | 92.4 | 13 | No |
| Intracranial hemorrhage | 5 | 47 | APL-like | 480.9 | 29 | No |
| Intracranial hemorrhage | 1 | 76 | APL-like | 55 | 23 | No |
| Pulmonary embolism secondary to DVT | 1 | 73 | APL-like | 195.3 | 26 | Yes |

DVT, deep venous thrombosis

**Table S5**. Baseline characteristics of AML *NPM1*^wt^ and *NPM1*^mut^ APL-like cohorts pre propensity score matching.

|  | ***NPM1*wt** | **APL-Like** | **P** |
| --- | --- | --- | --- |
| **Total** | n°100 | n°95 |  |
| **Age (years)** median (range) | 55.5 (45.25-64) | 66.42 (58-73) | **<0.001** |
| **WBC (x 10^9/L)** median (range) | 4.8 (2.24-17) | 42 (11.26-115.4) | **<0.001** |
| **Hb (g/dl)** median (range) | 9.2 (8.4-10.8) | 8.8 (7.5-9.9) | **0.013** |
| **Plt (x10^9/L)** median (range) | 56.5 (28.25-142) | 40 (24-82) | **0.013** |
| **LDH (U/L)** median (range) | 280 (202-485.5) | 463 (341-648) | **<0.001** |
| **D dimer (ng/ml)** median (range) | 881.5 (388.25-2047) | 5998 (2339-32239) | **<0.001** |
| **DD/FBG** median (range) | 2.20 (1.28-5.52) | 18.17 (5.74-302.68) | **<0.001** |
| **Vascular Complications** % (n°) | 6% (6/100) | 30.5% (29/95) | **<0.001** |
| **DIC Score** median (range)  **DIC score ≥ 4** % (n°) | 2 (0-3)  16% (16/100) | 4 (2-6)  57.9% (33/57) | **<0.001**  **<0.001** |

Differences between treatment groups were evaluated using Mann-Whitney test for continuous variables and Fisher exact tests or χ2 for categorical variables. Values in bold are statistically significant (P < 0.05). Abbreviations: WBC, white blood cell; Hb, hemoglobin; Plt, platelets; LDH, lactate dehydrogenase; FBG, fibrinogen; DD, D dimer; DIC, disseminated intravascular coagulation.

**Table S6**. Characteristics of patients post propensity score matching for age, leucocyte and platelets (matching method: exact).

|  | *NPM1*wt | APL-Like | *P* value |
| --- | --- | --- | --- |
| Post matching for age | n=74 | n=70 |  |
| Age (years) median (range) | 60 (52-65) | 63 (58-70) | **0.008** |
| WBC (x 10^9^/L) median (range) | 4.73 (2.24-19.93) | 52.24 (13.39-133.17) | **<0.001** |
| Hb (g/dl) median (range) | 9.1 (8.3-10.65) | 8.8 (7.48-9.75) | **0.031** |
| Plt (x 10^9^/L) median (range) | 75 (32-150) | 40 (25-67) | **0.001** |
| LDH (U/L) median (range) | 280 (201-481) | 518 (362-481) | **<0.001** |
| D dimer (ng/ml) median (range) | 1086 (516.5-2571) | 7170 (2476-27616) | **<0.001** |
| DD/FBG median (range) | 2.13 (1.33-6.1) | 16.93 (4.75-100) | **<0.001** |
| Vascular Complications % (n) | 4.1% (3/74) | 31.9% (22/69) | **<0.001** |
| DIC Score median (range)  DIC score ≥ 4 % (n) | 2 (0-3)  15.9% (11/69) | 4 (3-5)  28.3% (13/46) | **<0.001**  0.053 |
|  | ***NPM1wt*** | ***APL-Like*** | ***P value*** |
| Post matching for WBC | n=18 | n=16 |  |
| Age (years) median (range) | 56 (44.75-65) | 65.53 (55.13-76) | 0.080 |
| WBC (x 10^9^/L) median (range) | 2.34 (1.35-3.32) | 2.47 (1.48-4.16) | 0.568 |
| Hb (g/dl) median (range) | 8.9 (8.5-10.15) | 8.2 (7-9.96) | 0.103 |
| Plt (x 10^9^/L) median (range) | 54.5 (31.5-172.25) | 69 (40-120.25) | 0.932 |
| LDH (U/L) median (range) | 268.5 (200.7-431.3) | 252 (198-358) | 0.556 |
| D dimer (ng/ml) median (range) | 517 (297-1754) | 2066 (399-6875) | 0.161 |
| DD/FBG median (range) | 1.55 (1.04-4.84) | 6.1 (0.55-19.42)) | 0.162 |
| Vascular Complications % (n) | 5.6% (1/18) | 25% (4/16) | 0.164 |
| DIC Score median (range)  DIC score ≥ 4 % (n) | 2 (0-3)  16.7% (3/18) | 2 (1-4)  27.3% (3/11) | 0.099  0.646 |
|  | ***NPM1wt*** | ***APL-Like*** | ***P value*** |
| Post matching for platelets | n=47 | n=55 |  |
| Age (years) median (range) | 54 (45-63) | 66 (57.88-73) | **<0.001** |
| WBC (x 10^9^/L) median (range) | 7.96 (2.47-45.9) | 66.1 (57.87-73) | **<0.001** |
| Hb (g/dl) median (range) | 8.9 (8-10) | 8.9 (8-10) | 0.167 |
| Plt (x 10^9^/L) median (range) | 32 (24-48) | 32 (24-48) | 0.719 |
| LDH (U/L) median (range) | 347 (257-707) | 534 (384-702) | **0.026** |
| D dimer (ng/ml) median (range) | 1250 (465-3760) | 14453 (2722-35000) | **<0.001** |
| DD/FBG median (range) | 3.05 (1.24-9.27) | 34.57 (7.53-380) | **<0.001** |
| Vascular Complications % (n) | 10.6% (5/47) | 31.5 (17/54) | **0.015** |
| DIC Score median (range)  DIC score ≥ 4 % (n) | 3 (2-4)  27.7% (13/47) | 4 (3-5)  63.6 (21/33) | **<0.001**  **0.003** |

Differences between treatment groups were evaluated using Mann-Whitney test for continuous variables and Fisher exact tests or χ2 for categorical variables. Values in bold are statistically significant (P < 0.05). Abbreviations: WBC, white blood cell; Hb, hemoglobin; Plt, platelets; LDH, lactate dehydrogenase; FBG, fibrinogen; DD, D dimer; DIC, disseminated intravascular coagulation.

**Table S7.** Clinical, molecular and treatment characteristics in the training and validation (from AMLSG 09-09 clinical trial) cohorts.

|  | **Learning cohort** | | **Validation cohort** | | **P** |
| --- | --- | --- | --- | --- | --- |
|  | **n/median** | **range/%** | **n/median** | **range/%** |  |
| Total | 384 | - | 302 | - | - |
| Age (years) | 60.5 | (51-68) | 53.6 | (45.7-58.7) | **<0.001** |
| Male  Female | 175/384  209/384 | 45.6%  54.4% | 136/302  166/302 | 45.0%  55.0% | 0.94 |
| WBC (x 10^9^/L) | 35.1 | (10.5-87.9) | 21.8 | (6.1-55.4) | **<0.001** |
| Hb (g/dl) | 9.0 | (7.7-10.2) | 9.0 | (7.9-10.4) | 0.258 |
| Plt (x10^9^/L) | 52 | (31-90) | 77 | (47-126) | **<0.001** |
| LDH (U/L) | 616 | (385-1021) | 444.5 | (319-688.5) | **<0.001** |
| *FLT3* any  *TKD*  *ITD* | 195/379  40/379  169/379 | 51.5%  10.6%  44.6% | 91/302  40/302  55/302 | 30.1%  14.2%  18.2% | **<0.001**  0.344  **<0.001** |
| *IDH1-2* | 72/229 | 31.4% | 44/80 | 55.0% | **0.017** |
| *DNMT3A* | 98/201 | 48.8% | 167/302 | 55.3% | 0.437 |

Differences between treatment groups were evaluated using Mann-Whitney test for continuous variables and Fisher exact tests or χ2 for categorical variables. Values in bold are statistically significant (P < 0.05). Abbreviations: WBC, white blood cell; Hb, hemoglobin; Plt, platelets; LDH, lactate dehydrogenase; WHO, World Health Organization; TKD, tyrosine kinase domain; ITD, internal tandem duplication.

**Table S8.** Multivariate Analysis (Backward stepwise Cox Regression) for vascular events at 30 and 15 days in the validation cohort.

|  | **Covariates** | **HR (95% CI)** | **p value** |
| --- | --- | --- | --- |
| **Vascular events at 30 days** | Age (</≥ 60 y) | 1.32 | 0.31 |
|  | WBC (</≥ 50x10^9^/L) | 1.24 | 0.38 |
|  | Plt count (</> 50× 10⁹/L) | 0.57 (0.36-0.90) | **0.017** |
|  | APL-like phenotype | 1.48 | 0.089 |
| **Vascular events at 15 days** | Age (</≥ 60 y) | 1.20 | 0.64 |
|  | WBC (</≥ 50x10^9^/L) | 3.18 (1.7-6.0) | **0.0004** |
|  | Plt count (</> 50× 10⁹/L) | 0.58 | 0.11 |
|  | APL-like phenotype | 2.70 (1.4-5.1) | **0.0025** |

**Table S9.** Multivariate Analysis (Backward stepwise Cox Regression) for hemorrhagic events at 30 and 15 days in the validation cohort.

|  | **Covariates** | **HR (95% CI)** | **p value** |
| --- | --- | --- | --- |
| **Hemorrhagic events at 30 days** | Age (</≥ 60 y) | 1.31 | 0.32 |
|  | WBC (</≥ 50x10^9^/L) | 1.26 | 0.35 |
|  | Plt count (</> 50× 10⁹/L) | 0.61 (0.38-0.96) | **0.033** |
|  | APL-like phenotype | 1.49 | 0.09 |
| **Hemorrhagic events at 15 days** | Age (</≥ 60 y) | 1.21 | 0.62 |
|  | WBC (</≥ 50x10^9^/L) | 2. 98 (1.5-5.5) | **0.001** |
|  | Plt count (</> 50× 10⁹/L) | 0.58 | 0.11 |
|  | APL-like phenotype | 2.72 (1.4-5.2) | **0.002** |
